# Lamin A regulates chromatin structure and function in cardiac myocytes in a mouse model of lamin-cardiomyopathy

**DOI:** 10.1093/narmme/ugag046

**Published:** 2026-10-22

**Authors:** Priyatansh Gurha, Weiyue Wang, Benjamin Cathcart, Leila Rouhi, Ali J Marian

**Affiliations:** Center for Cardiovascular Genetics, Institute of Molecular Medicine and Department of Medicine, University of Texas Health Sciences Center at Houston, Houston, Texas 77030, United States; Center for Cardiovascular Genetics, Institute of Molecular Medicine and Department of Medicine, University of Texas Health Sciences Center at Houston, Houston, Texas 77030, United States; Center for Cardiovascular Genetics, Institute of Molecular Medicine and Department of Medicine, University of Texas Health Sciences Center at Houston, Houston, Texas 77030, United States; Center for Cardiovascular Genetics, Institute of Molecular Medicine and Department of Medicine, University of Texas Health Sciences Center at Houston, Houston, Texas 77030, United States; Center for Cardiovascular Genetics, Institute of Molecular Medicine and Department of Medicine, University of Texas Health Sciences Center at Houston, Houston, Texas 77030, United States

## Abstract

Mutations in the *LMNA* gene, encoding lamin A (LMNA), cause laminopathies. Cardiac involvement is common and a major cause of mortality and morbidity in laminopathies. LMNA interacts with chromatin through lamin-associated domains (LADs) and regulates gene expression. We report that LMNA deficiency increased genome-wide CTCF occupancy, predominantly outside of the constitutive LAD (cLAD) regions. CTCF occupancy was associated with increased gene expression and enrichment of double-stranded breaks (DSBs). LMNA deficiency had a modest effect on chromatin compartments but led to mergers, splitting, and shifting boundaries of one-third of the topologically associated domains (TADs) without affecting gene expression significantly. LMNA deficiency led to a gain of ∼600 and a loss of ∼1000 loops, which were associated with increased and decreased transcript levels, respectively. Reorganization of chromatin loops affected ∼25% of the differentially expressed genes in the LMNA-deficient cardiac myocytes. In multivariate analysis, CTCF-binding sites, chromatin loops, and cLADs were independent predictors of gene expression in the LMNA-deficient cardiac myocytes, whereas TADs and chromatin compartments were not. Thus, LMNA deficiency changes genome-wide CTCF occupancy and loop organization, which affect gene expression in cardiac myocytes. The findings provide new insights into the pathogenesis of cardiac involvement in laminopathies.

## Graphical Abstract

**Graphical Abstract:**
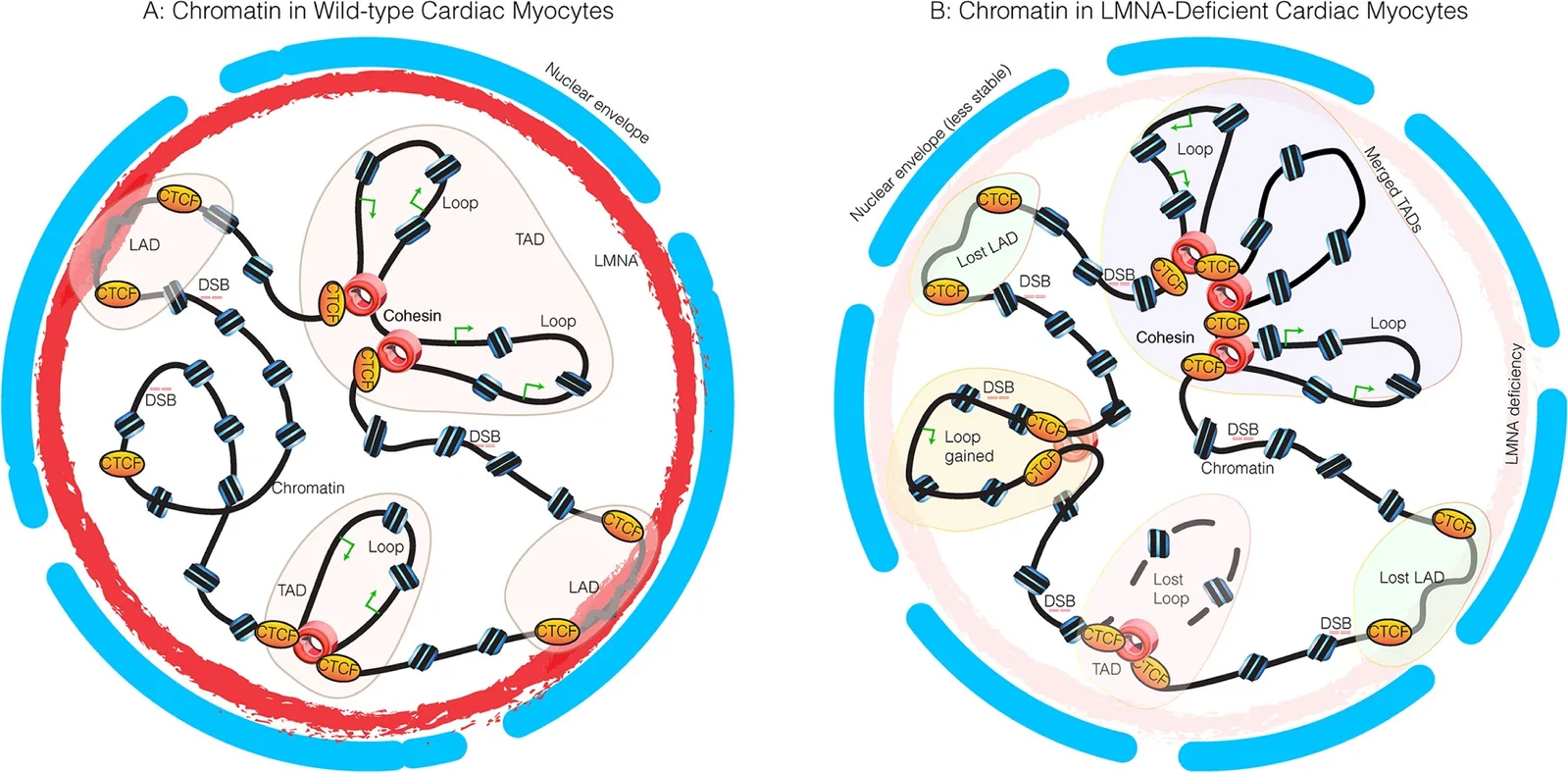

## Introduction

The spatial organization and structure of the mammalian genome is considered stochastic and dynamic [1]. Despite its dynamic nature, however, the genome is topographically and functionally organized into hierarchical spatial architectures, such as chromosome territories, compartments A and B, topologically associating domains (TADs), and chromatin loops [2]. Compartment A corresponds to euchromatin, is enriched in protein-coding genes with high transcriptional activity, and is preferentially located in the nuclear interior. This contrasts with compartment B, which is typically located at the periphery of the nucleus and comprises gene-poor heterochromatin with low transcriptional activity [3–5]. The B compartment is known to interact with the nuclear lamina [4].

At a finer scale, the genome is organized into self-interacting, typically sub-megabase-sized domains, which are referred to as TADs [1, 6]. TADs function to bring enhancers, promoters, and transcription factors into proximity to induce gene expression [1, 6]. Topologically associated domain (TAD) boundaries are enriched for CCCTC-binding factor (CTCF) and cohesin (SMC family of proteins) binding sites and are broadly conserved across mammalian species [7–10]. The convergently oriented CTCF motifs within TADs demarcate DNA segments that are extruded by the cohesin complex to form chromatin loops [11]. Chromatin loops play critical roles in genomic organization and regulation of gene expression, as they bring regulatory elements into proximity with their target promoters. Chromatin loops are substantially more dynamic and frequently rewired across cell types and during differentiation [11]. Regulation of gene expression through TADs and loops is implicated in various biological processes, including cell fate determination, proliferation, and differentiation [12]. However, how TADs and loops are regulated and whether and how they affect gene expression, particularly in pathological conditions, are largely unsettled questions.

The Lamin A (LMNA) protein, encoded by the *LMNA* gene, directly interacts with nucleosomes through its intrinsically disordered tail domain and retains the nucleosome at the periphery [13]. The interactions entail large genomic regions, which constitute lamin (or lamina)-associated domains (LADs) and comprise approximately 20%–30% of the genome in human and mouse cardiac myocytes and other cell types [4, 14, 15]. LADs are enriched at the nuclear periphery in conjunction with chromatin compartment B and exhibit features of heterochromatin with low transcriptional activity [4, 14, 16, 17]. LAD boundaries are enriched for insulator protein CTCF-binding sites, which fine-tune LAD border positioning [14, 18]. LMNA also interacts with CTCF and cohesins (SMC1A) at the TAD regions [19–21]. Thus, LMNA could contribute to genome organization through multiple mechanisms, including direct interactions with chromatin, peripheral tethering of chromatin through LADs, and contribution to higher-order genomic structures, such as TADs and chromatin loops [19–21].

Pathogenic variants in the *LMNA* gene cause a diverse array of diseases that are collectively referred to as laminopathies [22]. The phenotype arising in the heart is referred to as LMNA-cardiomyopathy and is characterized by progressive cardiac dilatation, cardiac dysfunction, conduction defects, and refractory heart failure [22, 23]. Several mechanisms are implicated in the pathogenesis of LMNA-cardiomyopathy, including altered gene expression, dysregulated epigenetic factors, DNA damage, activation of the DNA damage response (DDR) pathways, and changes in signaling pathways, among others [16, 17, 24–27]. At the chromatin level, pathogenic variants in the *LMNA* gene have been shown to disrupt interactions of LMNA with chromatin and lead to reorganization of LADs in human and mouse hearts and in inducible pluripotent stem cell-derived cardiomyocytes (iPSC-CMs) [16, 17, 27]. Altered interactions of the LMNA with peripheral chromatin in iPSC-CMs lead to aberrant expression of genes that are typically not expressed in myocyte lineage genes [27].

To determine the role of LMNA in chromatin structure and function in cardiac myocytes, we defined the genome-wide CTCF-binding sites and chromatin conformational states in the wild-type (WT) and a mouse model of LMNA-cardiomyopathy; the latter was generated upon the specific deletion of the *Lmna* gene in cardiac myocytes [16, 26, 28]. We integrated the findings on genomic compartments, TADs, chromatin loops, and genome-wide CTCF occupancy with data on gene expression, LADs, and DNA double-stranded breaks (DSBs) to gain insights into the molecular pathogenesis of LMNA-cardiomyopathy.

## Materials and methods

### Data access

All data are available from the corresponding authors upon request. The RNA-Seq, CUT&RUN, and Hi-C data have been deposited at GEO (RNA-Seq: GSE309726, END-Seq: GSE250321, LMNA CUT&RUN: GSE250321, CTCF CUT&RUN and Hi-C: GSE329699.

### Regulatory approval

The Animal Care and Use Committee of the University of Texas Health Science Center-Houston approved the use of mice in these studies (AWC-24-0020). The Guide for the Care and Use of Laboratory Animals, published by the US National Institutes of Health, was followed in conducting the studies.

### Anesthesia and euthanasia

Unless otherwise specified, mice were anesthetized with isoflurane inhalation (3% for induction and 0.5% to 1% for maintenance). Mice were euthanized by cervical dislocation after exposure to 100% CO_2_ inhalation.

### Sex as a biological variable

An equal number of male and female mice were used in these experiments. Sex was considered a biological variable and, whenever applicable, used in the statistical analysis to detect sex-dependent phenotypic effects.

### Wild-type (WT) and *Myh6-Cre:Lmna*^F/F^ mice

The phenotype in the WT and *Myh6*-*Cre:Lmna*^F/F^ mice has been published [16, 26, 28]. Mice (C57BL6/6J background) were matched for age (∼3 to 4 weeks old) and sex and were used in these experiments. Mice were housed in a vivarium with a 12-h light/dark cycle and were provided food and water *ad libitum*. Mice were genotyped by PCR, as published [16].

### Isolation of cardiac myocytes

Cardiac myocytes were isolated from mouse hearts upon collagenase perfusion using a Langendorff perfusion system, as published [16, 26, 28, 29]. In brief, mice were anesthetized with isoflurane and anticoagulated with heparin (200 units, IP). After explanting the heart, the aorta was cannulated and mounted on a Langendorff perfusion system. The heart was perfused with type 2 collagenase (2.4 mg/ml) at a flow rate of 4 ml/min until the heart became pale, spongy, and soft (∼5–10 min of perfusion). The heart was removed from the perfusion system and minced into small pieces in a 10% calf serum, 12.5 μM CaCl_2_, and 2 mM ATP buffer. The cardiac myocytes were dissociated from the minced tissue by gently pipetting up and down. The cell debris was removed by passing the dissociated cells through a 100 µm cell strainer. The isolated cells were left to precipitate by gravity in the presence of 2 mM ATP. The cardiac myocytes were separated from other cells upon centrifugation at 20 × *g* for a few minutes and were reintroduced to a stepwise gradient of CaCl_2_ ranging from 100 to 900 μM. The cardiac myocytes were treated with a lysis buffer to isolate the nuclei by centrifugation [16].

### Cleavage under targets & release using nuclease (CUT&RUN) assays

The genomic-binding sites of CTCF in cardiac myocytes were identified by the CUT&RUN assay, per a published protocol [16]. In brief, about one million cardiac myocytes were immobilized to magnetic beads, permeabilized, and incubated with an antibody against CTCF (Abcam #ab128873). The cells were then treated with Micrococcal Nuclease (MNase) and Protein A fusion protein. MNase was activated upon the introduction of Ca^2+^ to cleave the DNA at the binding sites of the antibody. The DNA was extracted from the DNA–protein complexes and used to construct libraries for massively parallel sequencing.

The CUT&RUN data were analyzed as published [16]. Data were visualized using the UCSC Genome Browser and the Integrative Genome Viewer [30, 31]. The sequence reads were mapped to the reference mouse genome GRCm39 using Bowtie2, and the peaks were called using MACS2 with the false discovery rate (FDR) set at *q* < 0.01 [32, 33]. The differential peaks were defined as the number of reads that were different between the WT and the LMNA-deficient genotypes at a *q-*value of < 0.05. A consensus peak set was built across all samples. Genomic intervals that overlapped by a peak in all 10 samples were classified as invariant, and those that overlapped in all 5 WT or all 5 *Myh6-Cre:Lmna*^F/F^ replicates were called as genotype-specific invariant peaks (Terminology is defined in Supplementary Table S1).

### High-throughput chromosome conformation capture (Hi-C)

Hi-C was performed on cardiac myocyte nuclei (*N* = 3 per genotype). In brief, freshly isolated cardiac myocytes were transferred to microcentrifuge tubes and pelleted by centrifugation at 500 × *g* for 5 min at 4°C. The supernatant was discarded, and cells were resuspended in ice-cold phosphate-buffered saline (1× PBS), followed by a second centrifugation at 500 × *g* for 5 min at 4°C. After complete removal of the supernatant, cell pellets were immediately snap-frozen in liquid nitrogen and stored at −80°C.

Samples were crosslinked and lysed using a sample preparation workflow according to the manufacturer’s instructions (Arima Hi-C Kit, A510008), followed by restriction enzyme digestion, biotinylation, and ligation. Ligated DNA was purified and sheared using a sonicator (Multi-Sample Sonicator, Active Motif, catalog #53130). The sheared DNA was then used for library preparation using a commercial library kit (Arima, A510008, A303011). Libraries were dual-indexed and amplified by PCR for 12 cycles, cleaned with 1× Solid-Phase Reversible Immobilization (SPRI) beads, quantified using a Qubit Fluorometer (Thermo Fisher Scientific) and TapeStation (Agilent). Samples were sequenced on an Illumina NovaSeq X platform with paired-end 150 bp reads up to 1B reads.

### Chromatin compartment analysis

The A and B compartments were defined using the Hi-C data at 100 kb resolution and the cooltools software [34]. In brief, the eigenvector (E1) was computed in each Hi-C library based on the Pearson correlation matrix of the observed/expected interaction frequencies, and oriented per-chromosome by correlating with the GC content (mouse genome assembly mm10). Bins were independently called A (E1 > 0) or B (E1 < 0) in each replicate. The high-confidence consensus compartment genomic regions were defined by retaining only bins that were concordant in all three replicates (3/3 consensus). Bins with discordant calls across replicates were left unassigned. A relaxed 2/3 consensus threshold was also analyzed, which yielded comparable results. Therefore, the stricter 3/3 consensus was used for all downstream analyses. The consensus bins were analyzed per genotype (WT and *Myh6-Cre:Lmna*^F/F^) and categorized into one of four compartment states: Stable, either in A or B compartment, when they were present in both genotypes, and shifted from A→B, or B→A in the *Myh6-Cre:Lmna*^F/F^ myocytes as compared to the WT genotype. Saddle plots were generated using cooltools (v0.5+), matrix computations were performed using NumPy, and visualization was rendered using Matplotlib in Python [35].

### TAD analysis

TADs were identified at 40 kb resolution using hicFindTADs (Hi-CExplorer) [36]. Insulation scores were computed using variable windows from 160 to 640 kb (step 40 kb), with boundaries called at a threshold of FDR ≤ 0.005 and a delta insulation score of ≥ 0.01. Domains smaller than 80 kb were excluded. Boundary calling was performed independently per replicate, and boundaries present in at least two of three replicates within 40 kb were identified, and their positions were averaged. A 3/3 consensus boundary list was also generated; however, it led to a smaller number of differential boundaries, and therefore, it was not used. Differential boundaries were identified within a 500 kb search window, and boundaries that were displaced ≥120 kb were classified as shifted, lost, or gained. TAD topology was classified as stable, shifted (at least one boundary was displaced by a minimum of 120 kb), merged (boundary between TADs was lost), or split (a TAD was divided into ≥ 2 in the *Myh6-Cre:Lmna*^F/F^ myocytes). Terminal TADs were excluded from all analyses. TAD sizes were compared using the Mann–Whitney *U* test. TAD calling and differential analyses were performed in Python using pandas, NumPy, and SciPy [35, 37].

### Chromatin loop analysis

Chromatin loops were called in each replicate using cLoop2 with a minimum of 20 paired-end tags (PETs) and an FDR ≤ 0.05 (binomial, Poisson, and hypergeometric tests) [38]. To determine the orientation of CTCF peaks, the convergent motifs were identified by retrieving the loop anchor sequences and scanning on both strands with the canonical CTCF matrix (JASPAR MA0139.1) and using log-odds scores against a uniform background. The best-scoring hit per anchor with a relative score ≥ 0.9 was retained.

For differential analysis, reads from the three WT and three *Myh6-Cre:Lmna*^F/F^ replicates were merged into a single contact matrix in each genotype. A union set of a total of 18 767 loops was assembled, and counts per loop were compared between the merged WT and merged *Myh6-Cre:Lmna*^F/F^ matrices using a Poisson test with Benjamini–Hochberg FDR correction. Loops were classified as gained (scaled fold change > 0, adj. *q* < 0.05) or lost (scaled fold change < 0, adj. *q* < 0.05) in *Myh6-Cre:Lmna*^F/F^, and the remaining loops were classified as not changed. To assess loop reproducibility across samples, per-replicate contact frequencies at differential loops were extracted from each of the six libraries individually and visualized as a row-scaled heatmap and as aggregate peak analysis (APA) pileups.

### LADs

LADs were defined as genomic regions that showed a significant enrichment of the *Lmna* reads over the background at a *q* < 0.01 [16]. The constitutive LADs (cLADs) were defined as regions that overlapped in at least three out of five samples. The loss-of-LAD (LoL) regions were defined as regions in the *Myh6-Cre:Lmna*^F/F^ myocyte genome that corresponded to the cLAD regions in the WT cardiac myocyte genomes. The LoL regions in the *Myh6-Cre:Lmna*^F/F^ myocyte genome were analyzed for chromatin features and CTCF-binding sites similar to the analysis performed in the WT cardiac myocyte genome.

### Data integration

To determine whether chromatin remodeling in LMNA-deficient cardiac myocytes affected gene expression, organization of LADs, and localization of DSBs, the Hi-C (*N* = 3 per genotype) and CTCF-binding site (*N* = 5 per genotype) data were integrated with published RNA-Seq (*N* = 5 per genotype, GSE309726), LADs (*N* = 5 per genotype, GSE250321), and DSB (*N* = 3 per genotype, GSE250321) data [16, 39]. All new and published experiments were performed in ∼3- to 4-week-old and sex-matched WT and LMNA-deficient cardiac myocytes.

The CTCF and cLAD peak coordinates were converted from the mouse reference genome assembly mm39 to mm10 using UCSC LiftOver. The cLAD intervals were merged into non-overlapping segments using GenomicRanges to prevent double counting [40]. The cLADs, CTCF peaks (±2 kb), and DSBs (±2 kb) were assigned to compartments, TADs, and loops using GenomicRanges. The fractions of cLADs, CTCF Peaks, and DSBs located in chromatin compartments, TADs, and loops were calculated. Enrichment relative to the genomic background was defined as the percentage of each feature divided by the percentage of the genome occupied by that feature (e.g. the percentage of CTCF peaks located in compartment A divided by the total number of CTCF peaks in the genome). Enrichment was also assessed using permutation tests with randomly distributed regions matched for size and number. Statistical significance was assessed by Fisher’s exact test comparing each chromatin category against all remaining called bins. Odds ratios and Benjamini–Hochberg-corrected *P*-values were reported.

Gene transcription start sites (TSSs) were extracted from GENCODE vM10 (mm10/GRCm38) and assigned to compartment, TAD, and Loop bins using GenomicRanges. For genes spanning multiple bins, the bin with the greatest overlap was used. Genes in uncalled bins or those that did not map to GENCODE vM10 were excluded. Genes were additionally classified as proximal or distal to CTCF peaks based on whether their TSS fell within ±2 kb of a CTCF peak. Gene expression (log2 of CPM) was compared between two categories using the Wilcoxon rank-sum test, and across multiple groups using the Kruskal–Wallis test with post-hoc pairwise Wilcoxon tests. All multiple comparisons were corrected using the Benjamini–Hochberg method in R. To determine the effect of an individual feature, a linear mixed model (|log₂FC| ∼ five binary feature indicators + mean WT log₂ CPM + (1 | chromosome); *n* = 11 953 expressed genes) was fit in R.

### Conventional statistics

The Gaussian distribution of the data was determined using the Shapiro–Wilk normality test. The t-test was used to compare the normally distributed data between two groups (genotypes). The Kruskal–Wallis test was used to compare data that departed from a Gaussian distribution. The categorical data were analyzed by Fisher’s exact or the Chi-square test. Statistical analyses were performed using GraphPad Prism 11.

## Results

### Phenotype in the *Myh6-Cre:Lmna*^F/F^ mice

The *Myh6-Cre:Lmna*^F/F^ mice exhibit cardiac dysfunction, cardiac arrhythmias, increased myocardial fibrosis, and excess cell death, as published [26, 28]. The phenotype is typically expressed starting from the third week of post-natal life, and the mice die within 4 weeks of age. The genomic experiments were performed at 18 days of age [26, 28].

### Genome-wide redistribution of CTCF-binding sites in the LMNA-deficient cardiac myocytes

Given that CTCF modulates interactions of LMNA with the genome, we determined the distribution of CTCF peaks in the WT and *Myh6-Cre:Lmna*^F/F^ cardiac myocytes by a CUT&RUN assay. There were six samples in each group. One IgG sample in the WT group had only 0.1 million reads, likely due to a failed reaction (Online Fig. 1 and Supplementary Table S2). Because CTCF peaks in each sample were called by comparing them to the IgG peaks in the corresponding sample, this sample was excluded from further analysis, to avoid overcalling CTCF peaks. Likewise, one CTCF sample in the *Myh6-Cre:Lmna*^F/F^ group had a very low number of peaks as compared to IgG peaks, indicating a lack of enrichment (Supplementary Fig. S1 and Supplementary Table S2). This sample was also excluded from further analysis. The final data were analyzed in five CTCF samples per genotype, along with the corresponding IgG for each sample.

**Figure 1.**
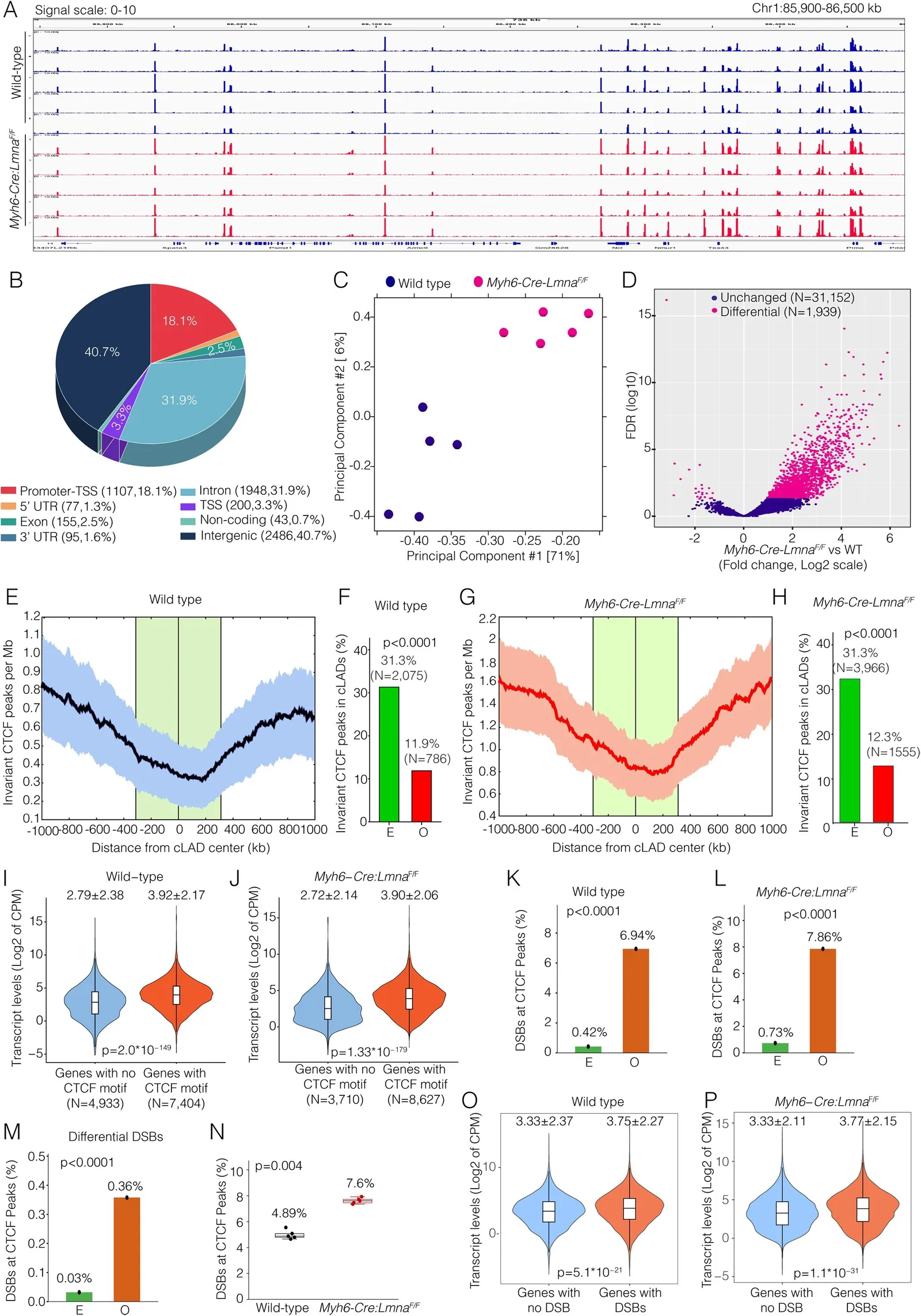
Characteristics of CTCF genomic-wide binding sites. (**A**) Genome browser tracks showing the CTCF peaks in 5 wild-type (WT) and 5 *Myh6-Cre:Lmna*^F/F^ (LMNA-deficient) cardiac myocytes. The selected regions depict the invariant peaks, which were present in all 10 samples. (**B**) Pie chart showing the annotation and genomic distribution of CTCF peaks across different genomic regions. (**C**) Principal component analysis (PCA) showing distinct separation of the CTCF peaks between the WT and *Myh6-Cre:Lmna*^F/F^ myocytes. (**D**) Volcano plot showing differential CTCF peaks between WT and KO. (**E–H**) Distribution frequency of the invariant CTCF peaks at the constitutive lamin-associated domains (cLADs) regions ± 1 million base pairs from the center of the cLADs, along with the quantitative data on the observed (O) and expected (E) CTCF Peaks in the WT and *Myh6-Cre:Lmna*^F/F^ myocytes. (**I** and **J**) Transcript levels of genes located at or near (±2 kb) the CTCF peaks in the WT and *Myh6-Cre:Lmna*^F/F^ myocytes. (**K** and **L)** Increased prevalence of DNA DSBs at the CTCF peaks in the wild-type and *Myh6-Cre:Lmna*^F/F^ myocytes (Observed versus Expected). (**M**) Increased prevalence of differential DSBs at the sites of CTCF peaks. (**N**) Percentage of DSBs located at the CTCF Peaks in the wild-type and *Myh6-Cre:Lmna*^F/F^ genotypes. (**O** and **P**) Transcript levels of genes located at or near (±2 kb) the DSB sites in the WT and *Myh6-Cre:Lmna*^F/F^ myocytes.

The number of pairwise sequence reads that were uniquely aligned to the mouse reference genome (GRCm39) in the WT and *Myh6-Cre:Lmna*^F/F^ myocytes were 30.1 ± 16.1 and 32.4 ± 4.0 million, respectively (Supplementary Table S2). The corresponding values in the IgG groups were 16.1 ± 8.6 and 17.2 ± 2.87 million reads, indicating about a 2-fold enrichment in the anti-CTCF antibody groups (Supplementary Table S2). Approximately 88% of the pairwise sequence reads in each genotype were uniquely aligned to the mouse reference genome (Supplementary Table S2). Representative CTCF peaks in 5 WT and 5 *Myh6-Cre:Lmna*^F/F^ cardiac myocyte genomes are shown in Fig. 1A. CTCF peaks were more numerous in *Myh6-Cre:Lmna*^F/F^ than in WT cardiac myocyte genomes (26 361.2 ± 8 278.4 versus 16 962.4 ± 4 472.2; *P* = 0.0280, Supplementary Table S2). The mean peak length was 329 and 359 nucleotides, corresponding to 0.21 ± 0.06% and 0.38 ± 0.15% in the WT and *Myh6-Cre:Lmna*^F/F^ cardiac myocyte genomes, respectively (*P *= 0.0199, Supplementary Table S2).

Approximately a fifth of the CTCF peaks (6624/29 798, 22.2%) were invariant in the WT genomes, defined as peaks that were detected in all five samples, whereas 12 663/43 883 (28.8%) of the CTCF peaks were invariant in the *Myh6-Cre:Lmna*^F/F^ cardiac myocyte genomes (Supplementary Fig. S2, Fisher’s exact test *P *< 0.0001). Approximately 13.1% of the CTCF peaks (6111/46 512) were invariant in all ten (5 WT and 5 *Myh6-Cre:Lmna*^F/F^) samples (Fig. 1A and Supplementary Fig. S3). The majority of the invariant CTCF peaks (4363/6659, 65.5%) identified in the cardiac myocyte genome in the present study also overlapped with the invariant CTCF peaks in the ENCODE datasets (Supplementary Fig. S4). Finally, about one-third of the peaks (13 760/46 512, 29.5%) were identified only in one sample (singleton, Supplementary Fig. S4).

The invariant CTCF peaks (present in all 10 samples) were analyzed for their genomic localization, which showed enrichment at the upstream and 5′ untranslated regions (5′ UTR), as compared to the expected number per a random distribution (Fig. 1B and Supplementary Table S3). A similar analysis for the singleton CTCF peaks, i.e. present only in one sample, showed enrichment at the upstream, 5′ UTR, and exons (coding sequence) regions (Supplementary Table S3).

Segregation of the CTCF peaks with the genotypes was analyzed by PCA, which showed a distinct separation of the CTCF peaks between the WT and *Myh6-Cre:Lmna*^F/F^ cardiac myocyte genomes, with principal component 1 accounting for 71% of the variance between the two genotypes (Fig. 1C). Comparison of all CTCF peaks between WT and *Myh6-Cre:Lmna*^F/F^ cardiac myocyte genomes identified 1939 differential peaks, of which 1917 peaks were increased and 22 decreased (Fig. 1D). The distribution of the invariant and all CTCF peaks that were differential between the two genotypes across genomic regions is depicted in Supplementary Table S4.

The findings suggest that LMNA deficiency is associated with an increased number of CTCF peaks in the cardiac myocyte genome and that a significant fraction of the CTCF peaks is invariant in the cardiac myocyte genome, regardless of the genotype.

### CTCF peaks are depleted in cLAD regions

To determine whether binding of LMNA to chromatin (LAD regions), influenced CTCF localization, the invariant CTCF peaks in the WT myocytes (*N* = 6624) were overlapped with the cLAD regions (*N* = 2572). The prevalence of CTCF peaks was lowest at or near the center of cLAD regions in the WT cardiac myocyte genome (Fig. 1E). Only 786/6624 (11.87%) invariant CTCF peaks were located at the cLAD regions as opposed to the expected 2075/6624 (31.3%) based on a random distribution (Fig. 1F, *P* = 1.89^–263^, permutation test). Similarly, when all CTCF peaks, whether variant or invariant, were considered, only 16.2% (*N* = 23 746/146 366) were in the cLAD regions, as opposed to the expected 31.3% under the assumption of a random distribution (*P *< 0.0001, Supplementary Table S5).

The *Myh6-Cre:Lmna*^F/F^ cardiac myocytes lack LMNA; therefore, the genomic regions corresponding to cLADs in the WT were designated as the LoL regions in *Myh6-Cre:Lmna*^F/F^. The prevalence of the invariant CTCF peaks in the *Myh6-Cre:Lmna*^F/F^ cardiac myocyte genome was lower in the LoL regions than the expected prevalence, as only about 12.3% of the invariant CTCF peaks were in the LoL regions as opposed to the expected 31.3% (Fig. 1, panels G and H).

Finally, we analyzed the localization of the CTCF peaks that were differential between the two genotypes to the LoL regions, which also showed a lower prevalence in the LoL regions (160/1939, 8.3%) than the expected prevalence (31.6%, *P* < 0.0001, Supplementary Fig. S5A). This was also the case when the analysis was restricted to the invariant CTCF peaks that were differential between the two genotypes (Supplementary Fig. S5B).

Thus, the findings show that CTCF peaks are less prevalent at the cLAD regions in cardiac myocytes, and the deletion of the *Lmna* gene does not increase the localization of the CTCF peaks to the LoL regions.

### CTCF peaks are associated with higher gene expression

To determine whether binding of CTCF to the chromatin affected gene expression, transcript levels of expressed protein-coding genes whose TSS were within ±2 kb of the CTCF peaks were compared with the transcript levels of genes outside this region in each genotype. Genes at the CTCF peaks (*N* = 7404) had higher transcript levels than those outside the CTCF peaks (*N* = 4933) in WT cardiac myocytes (Fig. 1I). Likewise, genes within the CTCF peaks (*N* = 8627) had higher transcript levels than those that were outside (*N* = 3710) in the *Myh6-Cre:Lmna*^F/F^ cardiac myocytes (Fig. 1J). The higher transcript levels of genes at the CTCF motifs were consistently observed in all 10 samples, regardless of the genotype, reflecting biological consistency and reproducibility of the finding (Supplementary Fig. S6). Moreover, transcript levels of genes located at the invariant CTCF peaks were also higher than those located outside, both in the WT as well as in the *Myh6-Cre:Lmna*^F/F^ cardiac myocytes (Supplementary Fig. S7). Thus, the findings, which are consistent across samples, show that binding of CTCF to chromatin is associated with enhanced gene expression.

### DSB are enriched at CTCF peaks

Given that CTCF is implicated in DSB repair and because the CTCF peaks were enriched in cLAD boundaries, we analyzed co-localization of the CTCF peaks and DSBs in cardiac myocyte genomes [41]. Approximately 7% of all DSBs were located at the CTCF peaks in the WT cardiac myocyte genome, as opposed to the expected 0.42% based on genomic occupation of the CTCF peaks, which is a 16.5-fold enrichment (*P* < 0.0001, Fig. 1K). A similar level of enrichment was also observed in the *Myh6-Cre:Lmna*^F/F^ myocyte genome, as ∼8% of the DSBs were localized to the CTCF peaks, as opposed to the expected 0.73%, a 10.8-fold enrichment (*P* < 0.0001, Fig. 1L). Concerning the differential DSBs (*N* = 6772), 0.36% of the differential DSBs were located at the differential CTCF peaks (*N* = 1939), as opposed to the expected 0.03%, which is ∼12-fold enrichment (Fig. 1M, *P* < 0.0001). Consistent with these findings, the CTCF peaks in the *Myh6-Cre:Lmna*^F/F^ cardiac myocyte genomes were ∼1.6 times more likely to contain a DSB than the CTCF peaks in the WT genomes (*P* = 0.004, Fig. 1N).

In agreement with the association of CTCF peaks with increased transcript levels and the preponderance of DSBs at the CTCF peaks, transcript levels of genes containing DSBs were higher than genes that did not contain a DSB, both in WT and *Myh6-Cre:Lmna*^F/F^ myocyte genomes (Fig. 1, panels O and P, respectively). Collectively, the data show that DSBs are more prevalent at the CTCF-binding sites. Given that CTCF peaks are associated with increased transcript levels, the findings imply coupling of DSBs to gene expression (transcription-coupled DSBs) in the cardiac myocyte genome.

### LMNA deficiency is associated with a modest effect on genomic compartments

Despite the dynamic nature of chromatin positioning, the genome is organized into A and B compartments, corresponding to central euchromatin and peripheral heterochromatin, respectively [3, 42]. To determine whether LMNA deficiency affected chromatin compartmentalization, Hi-C data were analyzed to identify the A and B compartments in WT and *Myh6-Cre:Lmna*^F/F^ cardiac myocyte genomes. Saddle plots for chromatin compartmentalization showed a similar profile in both genotypes, with compartment A being the predominant compartment (Fig. 2A). A total of 13 343 protein-coding genes were expressed in cardiac myocytes, of which 11 089 (83.1%) were assigned to a compartment. The remaining 2254 were excluded from analysis because either their gene symbols were absent from the GENCODE vM10 database or they were not present in 3/3 replicates. Genes in compartment A (*N* = 10 106) had higher transcript levels than those in compartment B (*N* = 983) both in WT and *Myh6*-*Cre:Lmna*^F/F^ myocytes (Fig. 2B). Among the protein-coding genes in compartment A, 86.6% (71.3% considering all biotypes) had at least 1 copy per million transcripts (CPM) in all 5 WT samples. In contrast, 72.6% of protein-coding genes in compartment B were completely silent (82.1% across all biotypes), defined as having zero CPM in all five wild-type replicates (Supplementary Fig. S8).

**Figure 2.**
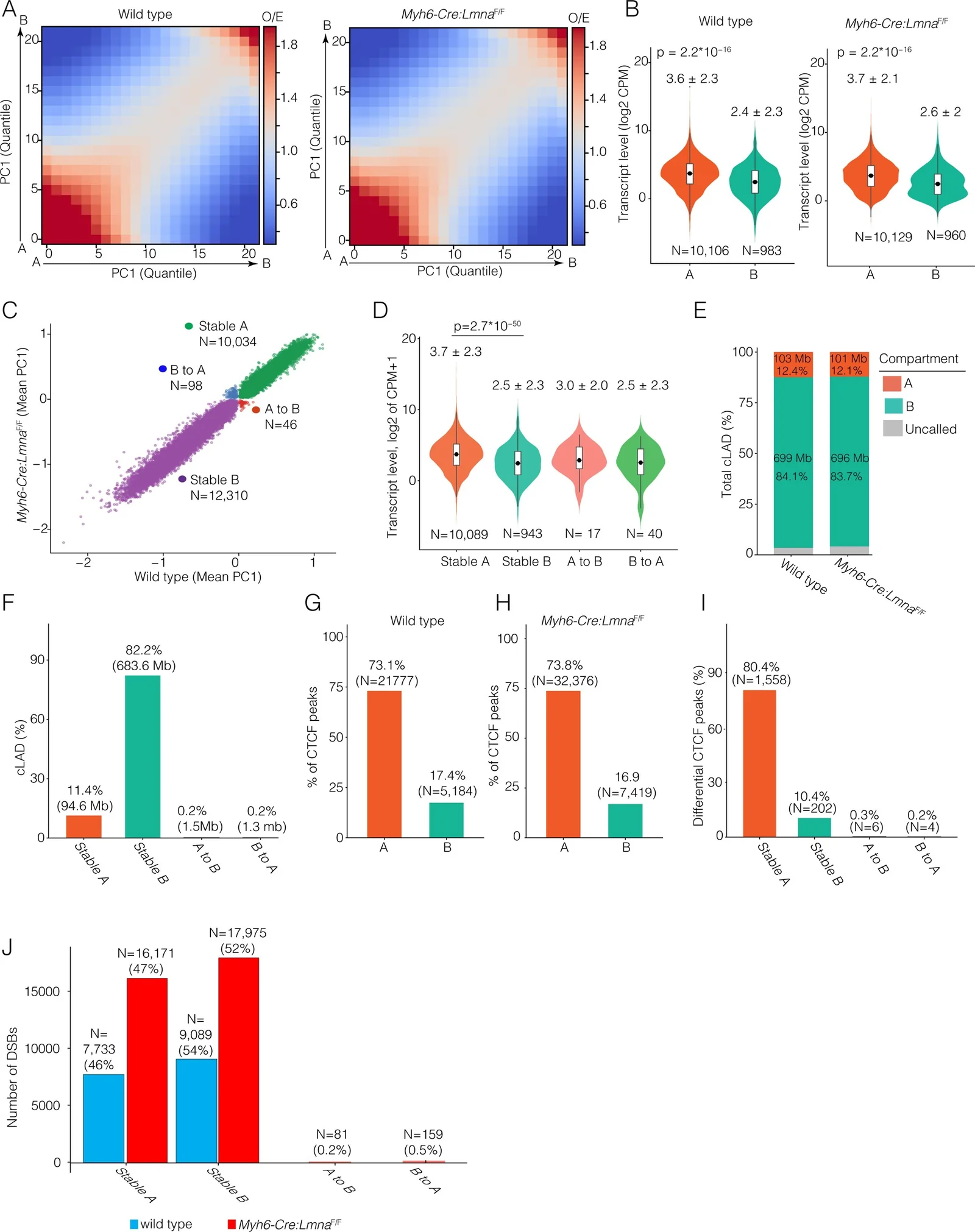
Characteristics of chromatin compartments. (**A**) Saddle plots visualizing chromatin A and B compartments in the WT and *Myh6-Cre:Lmna*^F/F^ myocytes. (**B**) Violin plots depicting transcript levels of genes located in the A and B compartments in each genotype. (**C**) Regression plot depicting the number of stable and switched compartments A and B in the *Myh6-Cre:Lmna*^F/F^ as compared with the WT myocytes. (**D**) Transcript levels of genes located in stable and switched compartments. (**E**) Total number and percentages of cLADs that overlap with compartments A and B in the WT and *Myh6- Cre:Lmna*^F/F^ myocytes. (**F**) Percentage of the cLADs located in the stable and shifted chromatin compartments. (**G** and **H**) Number and percentage of CTCF peaks located in the A and B components in the WT and *Myh6-Cre:Lmna*^F/F^ myocytes. (**I**) Number and percentages of the differential CTCF peaks located in the stable and shifted chromatin compartments. (**J**) The number and percentages of the DNA DSBs at stable and shifted chromatin compartments.

Deletion of the *Lmna* gene was associated with repositioning of a small fraction of the compartments (144/22 488, 0.64%), comprising 98 that were switched from B to A and 46 from A to B (Fig. 2, panel C). Whereas transcript levels of genes located in stable A (*N* = 10 089) were higher than those located in the stable B (*N* = 943) compartment, the switch in the compartment did not significantly affect gene expression as the transcript levels of 17 genes that were switched from A to B and 40 genes that were switched from B to A were not different between the WT and *Myh6-Cre:Lmna*^F/F^ myocytes (Fig. 2E). A similar pattern was observed when the analyses were repeated with the DEGs, which had higher transcript levels in the A than B compartment in both genotypes (Supplementary Fig. S9A). Likewise, transcript levels of genes at stable (unchanged) A (*N* = 5 110) were higher than those in the stable B (*N* = 481) compartment (Supplementary Fig. S9B). Moreover, there were no differences in the transcript levels of the small fraction of the DEGs that had switched compartments (Supplementary Fig. S9B).

To determine whether deletion of the *Lmna* gene affected localization of cLADs to chromatin compartments, cLAD regions were first mapped to genomic compartments in the WT cardiac myocyte genome. Approximately 84% of the cLAD regions were located in the B compartment (Fig. 2E). LMNA deficiency did not affect the localization of the LoL regions, corresponding to the cLAD regions in the WT genome, to the genomic compartments in the *Myh6-Cre:Lmna*^F/F^ genomes (Fig. 2E). Likewise, approximately 82% and 11.4% of the cLADs were in the stable (unchanged) compartments B and A, respectively (Fig. 2F). Only 1.5 Mb (0.2%) and 1.3 Mb (0.2%) of the cLAD segments were switched from A to B and B to A compartments, respectively (Fig. 2F). Although the number of compartmental switches was small, there was a higher number of A-to-B switches than B-to-A switches in the LMNA-deficient cardiac myocyte genome (16/46, 34.8% versus 14/98, 14.3%; Fisher’s exact test, *P* < 0.01).

To determine whether LMNA deficiency affected the localization of CTCF to the genomic compartments, the number of CTCF peaks in each compartment was determined. The CTCF peaks were mainly (>73%) in the A compartment in both genotypes (Fig. 2, panels G and H). Likewise, ∼80% of the differential CTCF peaks were in the stable A compartment and ∼10% in the stable B compartment (Fig. 2I). The LMNA deficiency had a negligible effect on the localization of CTCF peaks to the compartments, as only ∼0.5% of the consensus peaks were shifted between the two compartments (Fig. 2I).

Finally, to determine whether LMNA deficiency was associated with changes in the localization of the DSBs to A and B compartments, the frequency of the DSBs in each genomic compartment was determined and compared between WT and *Myh6-Cre:Lmna*^F/F^ myocytes. Although DSBs were more common in the LMNA-deficient myocyte genome, approximately half of the DSBs in each genotype were in the A compartment and another half in compartment B (Fig. 2J). Only ∼0.2% of the DSBs had shifted from A to B and another 0.5% from B to A in the LMNA-deficient cardiac myocyte genome (Fig. 2J).

Overall, the data suggest that LMNA deficiency had a modest effect on genomic compartmentalization and did not affect transcript levels of genes whose location in the genomic compartment was changed. Furthermore, LMNA deficiency did not affect the localization of CTCF peaks and DSBs to the genomic compartments. In the absence of LMNA, however, the LoL regions were more likely to shift from the A to B compartment.

### LMNA deficiency is associated with destabilization of TADs in cardiac myocytes

To determine whether LMNA deficiency affected TAD formation, we determined the TAD boundaries and identified a total of ∼3000 boundaries in each genotype, of which 2806 and 2729 boundaries were present in three independent WT and *Myh6-Cre:Lmna*^F/F^ samples, respectively, and were considered consensus boundaries (Fig. 3A). Likewise, 2785 and 2708 TAD domains, defined as regions between two boundaries, were identified in the WT versus *Myh6-Cre:Lmna*^F/F^ myocytes, respectively (Fig. 3B). The median TAD sizes were similar between the two genotypes (540 and 600 kb in the WT and *Myh6-Cre:Lmna*^F/F^ myocyte genomes, respectively, *P* = 0.07, Fig. 3C). The boundaries of 31.2% (*n* = 870) of the TADs were changed in the *Myh6-Cre:Lmna*^F/F^ cardiac myocyte genomes, while 68.8% (*n* = 1915) remained stable (Fig. 3D). The changes involved the merger of two or more TADs (59.4%), which was the most common, splitting of a TAD (30.1%), and boundary shifting (10.5%) (Fig. 3E). Representative Hi-C contact heatmaps illustrating examples of merger, splitting, and boundary shift are shown in Fig. 3F. The shift in TAD boundaries led to the repositioning of the TSS of 2 054 DEGs, comprising 1034 upregulated and 1020 downregulated genes in the *Myh6-Cre:Lmna*^F/F^ cardiac myocytes, whereas 3396 DEGs were in the unchanged TADs (Fig. 3G). The changes in TAD organization did not change transcript levels of the affected genes (Fig. 3H).

**Figure 3.**
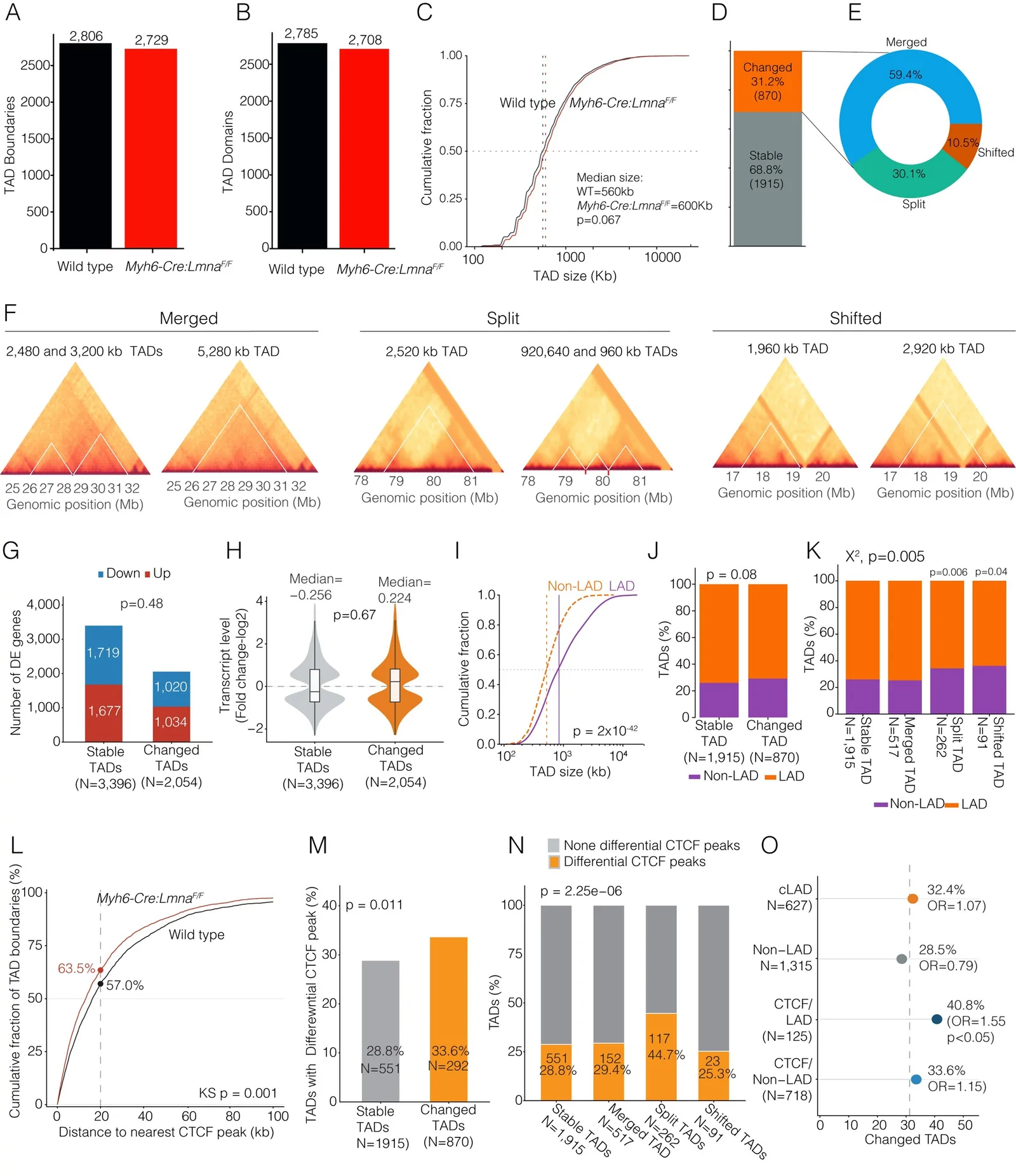
Characteristics of TADs. (**A**) The number of TAD boundaries in the wild-type (WT) and *Myh6-Cre:Lmna*^F/F^ myocytes. (**B**) The number of TADs in the WT and *Myh6-Cre:Lmna*^F/F^ myocytes. (**C**) Distribution of TAD size in the WT and *Myh6-Cre:Lmna*^F/F^ myocytes. (**D**) Percent of stable and shifted TADs in *Myh6-Cre:Lmna*^F/F^ compared with WT myocytes. (**E**) Categories of shifted TADs. (**F**) Examples of rearranged TADs, comprising a merged TAD, a split TAD, and a shifted TAD. (**G**) The number of DEGs in the stable and changed TAD regions. (**H**) Transcript levels of the DEGs at the stable and changed TAD regions. (**I**) Distribution of the TAD size in the cLAD and non-LAD regions. (**J**) Distribution of the stable and changed TADs in the cLAD and non-LAD regions. (**K**) Distribution of the stable, merged, split, and shifted TADs in the cLAD and non-LAD regions. (**L**) Localization of the TADs and CTCF peaks in the WT and *Myh6-Cre:Lmna*^F/F^ myocytes. (**M**) Percent of stable and changed TADs that contained differential CTCF peaks. (**N**) Percent of TADs with and without differential CTCF peaks at the stable, merged, split, and shifted TAD regions. (**O**) Combinatorial effects of CTCF and cLAD on changing TADs.

LADs are considered the structural backbone that anchors the genome to the nuclear envelope and thereby guides its overall folding [4]. To determine whether cLADs were involved in defining TAD boundaries, cLADs were overlapped with TADs. Overall, the TADs in the cLAD regions were larger in size than those that were outside in the WT cardiac myocyte genome (median 840 versus 520 kb, *P* = 2 × 10⁻⁴²; Fig. 3I). There was a trend toward localization of changed TADs to the cLAD regions compared with unchanged TADs (*P* = 0.08; Fig. 3J). When analyzed by topology class, both split TADs (34.4% versus 26.0%; *P* = 0.006) and shifted TADs (36.3% versus 26.0%; *P* = 0.04) were significantly enriched in the cLAD regions, whereas merged TADs were not (25.3% versus 26.0%; *P* = 0.78) (Fig. 3K).

To determine whether CTCF, which forms the structural anchors of TADs, contributes to TAD reorganization, the localization of TAD boundaries to CTCF peaks (within 20 kb) was analyzed in both genotypes. Approximately 57.0% of the TAD boundaries in the WT cardiac myocyte genome had at least one CTCF peak as compared with 63.5% in *Myh6-Cre:Lmna*^F/F^ myocytes (*P* = 0.001, Fig. 3L). Approximately 70% of CTCF peaks were located within 40 kb of a TAD boundary (Fig. 3L). Likewise, a greater percentage of the differential CTCF peaks were at the changed TAD sites in the *Myh6-Cre:Lmna*^F/F^ myocytes compared with stable TAD sites (33.6% versus 28.8%, *P* = 0.011, Fig. 3M). This enrichment was most pronounced at TADs that underwent splitting in the *Myh6-Cre:Lmna*^F/F^. Approximately 44.7% (117/262) of split TADs harbored differentially bound CTCF peaks, compared to 28.8% (551/1915) of the stable TADs, 29.4% (152/517) of the merged TADs, and 25.3% (23/91) of the shifted TADs (Fig. 3N, *P* = 2.25 × 10⁻⁶). Finally, the contributions of CTCF occupancy and cLAD localization to TAD reorganization were analyzed by univariate logistic regression. TADs that contained CTCF peaks and were in the cLAD region (CTCF+/cLAD) were more likely to be changed (odds ratio = 1.55, *P* < 0.05), whereas cLAD alone did not change the TAD structure (Fig. 3O).

To test whether TAD remodeling was associated with the localization of DSBs, END-seq peaks (±2 kb) were intersected with the TAD regions. Overall, DSB density was similar between stable and changed TADs in the WT (7.52 versus 7.63 peaks/Mb, respectively, *P* = 0.65) and in the *Myh6-Cre:Lmna*^F/F^ myocytes (15.42 versus 15.20 peaks/Mb, respectively, *P* = 0.57; Supplementary Fig. S10). Differential DSB peaks were equally distributed between stable and changed TADs (2.73 versus 2.58 peaks/Mb, respectively, *P* = 0.96, Supplementary Fig. S10). DSB densities in merged, split, and shifted TADs were also comparable to those in stable TADs (*P* = 0.73; Supplementary Fig. S10).

The findings suggest that LMNA deficiency affects the organization of a third of the TADs, mainly leading to the merger of two or more TADs; however, TAD remodeling does not appear to be an independent predictor of transcriptional changes in LMNA-deficient cardiac myocytes.

### LMNA deficiency is associated with reorganization of chromatin loops

To determine whether LMNA deficiency affected chromatin loop organization, loops were identified and compared between the WT and *Myh6-Cre:Lmna*^F/F^ cardiac myocyte genomes. LMNA deficiency (*Myh6-Cre:Lmna*^F/F^) resulted in the loss of 1029 and the gain of 603 chromatin loops (Fig. 4A). Gain and loss of loops were consistent among the three replicates in each genotype (Fig. 4B and Supplementary Fig. S11). Representative examples of gained and lost loops are shown in Fig. 4C and D. Normalized interaction scores and aggregate loop-strength enrichment scores showed that gained loops had higher enrichment in the *Myh6-Cre:Lmna^F^*^/F^ than in WT myocytes, lost loops had the opposite pattern, and stable loops showed no enrichment (Fig. 4E and Supplementary Fig. S12A). Gained loops were significantly shorter than the lost loops (median 180 kb versus 239 kb; *P* < 0.0001), with both categories spanning a broad size range (Supplementary Fig. S12B).

**Figure 4.**
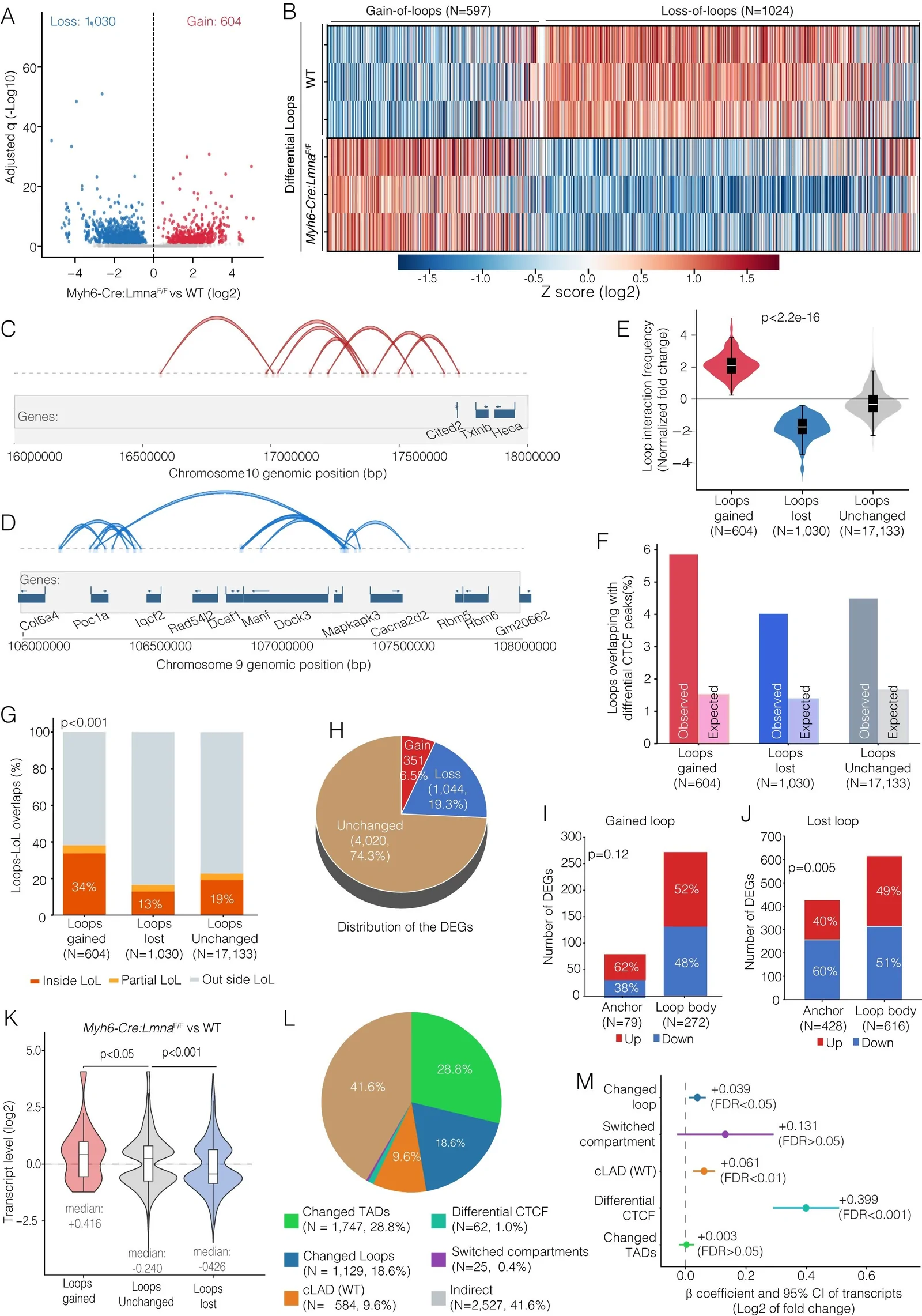
Characteristics of chromatin loops. (**A**) Volcano plot showing the number of gained and lost loops in the *Myh6-Cre:Lmna*^F/F^ compared with the WT myocytes. (**B**) Heat map of gained and lost loops in three biological replicates per genotype. (**C**) An example of a loop gained. (**D**) An example of a loop lost. (**E**) Strength of interactions at the gain, loss, and unchanged loop regions per genotype. (**F**) Distribution of all CTCF peaks at the loops in the WT and *Myh6-Cre:Lmna*^F/F^ myocytes. (**H**) Distribution of the observed differential CTCF peaks versus expected at the gain, loss, and unchanged loops. (**G**) Localization of loops at the loss of LAD (LoL) regions at the gain, loss, and unchanged loop regions. (**H**) Distribution of the differentially expressed genes (DEGs) at the gain, loss, and unchanged loops. (**I**) Number and percentage of the DEGs at the anchor and body of the gained loops. (**J**) Number and percentage of the DEGs at the anchor and body of the lost loops. (**K**) Transcript levels of DEGs located at the gain, unchanged, and loss of loop regions (**L**) Distribution of the DEGs at the gain or loss of loops, LADs, changed TADs, and switched compartments. (**M**) Combinatorial effects of chromatin changes in the chromatin on DEGs in *Myh6-Cre:Lmna*^F/F^ myocytes, showing changed loops, cLADs, and differential CTCF exerting independent effects.

To determine whether CTCF occupancy at loop anchors (start and end of loops) was altered in *Myh6-Cre:Lmna*^F/F^ myocytes, CTCF peaks were overlapped with loop anchors (average of three samples). In WT myocytes, 68% of loops had CTCF peaks at both anchors, 27% at one anchor, and 5% at neither; in *Myh6-Cre:Lmna*^F/F^, these proportions shifted to 85%, 11%, and 4%, respectively (*P* < 0.00005; Supplementary Fig. S13). Only a small subset of differential CTCF peaks overlapped with the loop anchors (<6%). Nevertheless, the differential CTCF peaks were more common at the gained loop anchors than expected (Fig. 4F, OR = 1.33, *P* = 0.029, Fisher’s exact test). Among loops that had a canonical motif at both anchors, the convergent orientation was the most common configuration in all three classes of gained, lost, and unchanged loops (50.0%, 58.2%, and 54.2%, respectively), and the divergent configuration was the least common (6.7%–9.7%, Supplementary Fig. 12B).

To determine whether LoL in the *Myh6-Cre:Lmna*^F/F^ myocytes affected loop formation, loops were overlapped with the LoL regions. Approximately 34% of the gained loops were within or partially overlapped with the LoL regions, as opposed to 13% of the lost loops and 19% of the unchanged loops (*P* < 0.001; Fig. 4G). The finding suggests that de novo loop formation in the LMNA-deficient myocytes predominantly occurs at chromatin located in the LoL regions.

To determine whether DSBs were more common in the chromatin loops, DSB peak density (peaks/Mb) at the loop regions was compared to the genome-wide expected density in each genotype. DSB peaks were modestly but significantly enriched at the loops in the WT (Supplementary Fig. S14, *P* < 0.001). In contrast, the DSB density was slightly reduced in the loop regions in the *Myh6-Cre:Lmna^F/F^* cardiac myocytes (Supplementary Fig. S14, *P* < 0.001). The findings suggest a propensity of DSB localization to the chromatin loops. However, the density of the differential DSBs was comparable across loop categories (Unchanged, 3.21; Gain, 3.40; Loss, 3.58 peaks/Mb), with no significant differences between the genotypes (*P* = 0.347 and *P* = 0.405, respectively, Supplementary Fig. S14). The findings suggest that gained and lost loops do not show preferential accumulation of DSBs relative to unchanged loops.

To assess the transcriptional effects of loop remodeling, DEGs were mapped to the loop regions. Approximately 1 400 DEGs were localized to the differential loop regions, comprising 351 upregulated and 1044 downregulated DEGs (Fig. 4H). The DEGs were analyzed according to gain or loss of loops, which showed that 62% of the 79 DEGs at the gained loop anchors were upregulated, and 38% were downregulated (Fig. 4I). Likewise, 52% of the 272 DEGs at the loop body were upregulated, and 48% were downregulated (*P *= 0.12, Fig. 4I). A similar analysis at the loss of loop regions showed that 60% of the 428 DEGs at the anchor regions and 51% of the 616 DEGs at the loop body regions were downregulated (*P *= 0.005, Fig. 4J). Consistent with these findings, transcript levels of DEGs at the gained loop regions were higher than those located at the lost loop regions or unchanged loops (Fig. 4K). Gain and loss of chromatin loops in the LMNA-deficient cardiac myocytes involved ∼25% of the DEGs, which were involved in metabolism, cardiac contraction, and myocyte action potential, among others (Supplementary Fig. S15).

Finally, the combinatorial effects of CTCF, cLADs, TADs, and loops on gene expression were determined, which showed that 58.4% of the DEGs in the *Myh6-Cre:Lmna*^F/F^ mouse cardiac myocytes were localized to one of the above structural changes in the chromatin, with TADs comprising the largest number (1747, 28.8%) of the DEGs (Fig. 4L). Because of the collinearity of the selected chromatin features, multivariate mixed model analysis was performed, which showed that differential CTCF peaks, cLADs, and changed loops had significant independent effects on the expression of the DEGs, with the differential CTCF exerting the largest effect size (Fig. 4M).

The findings collectively suggest that LMNA deficiency is associated with re-organization of loops, and the changed loops along with the differential CTCF peaks and cLADs were independently associated with gene expression.

## Discussion

Laminopathies comprise over a dozen distinct diseases, including cardiomyopathies, which are a prominent feature and a major cause of death [22, 43]. How mutations in the *LMNA* gene cause such a diverse array of phenotypes remains partially unsolved. To gain insight into the molecular pathogenesis of cardiac involvement in laminopathies, we focused on the well-established interactions of the LMNA with the chromatin and characterized the role of LMNA protein in genomic organization and functions in cardiac myocytes at two levels: First, we delineated the impact of the LMNA deficiency on genomic occupancy of CTCF, a multi-functional protein that plays a major role in genomic organization and function; Second, we defined the chromatin conformational changes by Hi-C in the WT (LMNA-competent) and LMNA-deficient (*Myh6-Cre:Lmna*^F/F^) cardiac myocytes [18, 20, 44].

The findings highlight the major role of the LMNA in regulating genomic structure and functions, as the changes in chromatin structure affected the expression levels of about half of the DEGs in the *Myh6-Cre:Lmna*^F/F^ cardiac myocytes. The effects of LMNA on gene expression were mediated through several genomic changes, including redistribution and an increased number and width of the CTCF peaks in the LMNA-deficient cardiac myocytes. Consequently, the differential CTCF peaks between the WT and *Myh6-Cre:Lmna*^F/F^ mice were almost exclusively detected in the LMNA-deficient cardiac myocytes. The increased genomic occupancy of CTCF in cardiac myocytes was functionally significant, as the transcript levels of genes located at or near the CTCF peaks were higher than those that were elsewhere.

The mechanism(s) by which LMNA-deficiency affects the affinity or recruitment of CTCF to chromatin in cardiac myocytes were not explored. The reduced number of CTCF peaks at the cLAD regions and their enrichment at LAD boundaries in the WT myocytes suggest that LMNA may limit or hinder CTCF binding to the cLADs in the genome, which has been observed previously [18]. However, despite the deletion of the *Lmna* gene, the CTCF peaks remained less abundant in the LoL regions in the *Myh6-Cre:Lmna*^F/F^ myocytes, likely because of persistence of an intrinsic chromatin state that is not permissive for CTCF binding even after the loss of LMNA. For example, cLADs are enriched in repressive histone marks such as H3K9me3, H3K27me3, and CpG methylation, which may reduce chromatin accessibility or interfere with CTCF occupancy [17, 45]. Nevertheless, the exact nature of putative DNA modifications at the cLAD regions that hinder binding of CTCF to chromatin remains to be determined.

A notable finding was marked enrichment of DSBs at or near the CTCF peaks in the LMNA-deficient cardiac myocytes, which is consistent with the co-localization of CTCF and topoisomerase 2B, which generate the DSBs [46]. In addition, given the role of CTCF in gene expression, the increased number of DSBs at the CTCF motifs implies coupling of the DSBs to gene expression [47, 48]. In agreement with the role of transcription in DSB generation, DSBs were more abundant at the transcription start sites and less frequent in the cLAD regions in the WT myocytes; the latter is associated with suppressed gene expression in cardiac myocytes as well as other cells [17, 47, 48]. Likewise, DSBs were increased in the LMNA-deficient cardiac myocytes, particularly at the LoL regions [16]. Collectively, the data imply that DSBs are coupled to transcription at the CTCF motifs in cardiac myocytes, as in other cell types [17, 47, 48].

Although chromatin A and B compartments could vary between cell types as genes shift between active and repressive chromatin environments, approximately 91% of the coding genes in cardiac myocytes were in compartment A, which is consistent with the enrichment of these genes in the euchromatin region in human and other genomes [49, 50]. LMNA, which anchors chromatin to the nuclear periphery, had a modest effect on chromatin compartment remodeling in cardiac myocytes.

The loss of LMNA in *Myh6-Cre:Lmna*^F/F^ myocytes affected the structural organization of about one third of the TADs in the cardiac myocyte genome, mainly leading to the merger of two or more TADs. The merger of TADs, which was associated with increased CTCF motifs, along with changes in TAD boundaries, involved re-localization of ∼40% of the DEGs between the WT and *Myh6-Cre-Lmna*^F/F^ myocytes. However, the changes in TADs were not associated with changes in gene expression.

Chromatin loops are mainly organized by CTCF and cohesins [11, 51, 52]. LMNA deficiency was associated with reorganization of loops and the formation of de novo loops predominantly at the LoL regions, which are defined as genomic regions in the *Myh6-Cre:Lmna*^F/F^ myocytes that correspond to cLADs in the WT genome. Consistently, many of the gained loops lacked CTCF peaks, in contrast to stable loops, which suggests that LMNA deficiency makes the LoL regions more permissive to looping, likely through mechanisms independent of CTCF. Gain and loss of loops involved ∼25% of the DEGs, which included genes involved in metabolism, mitochondrial oxidative phosphorylation, cardiac contraction, and cardiac rhythm. Alterations in these biological pathways are in accord with the observed cardiac dysfunction, cardiac arrhythmias, impaired oxidative phosphorylation, and cardiac metabolism observed in laminopathies [24, 53, 54].

Overall, changes in the organization of the chromatin in the *Myh6-Cre:Lmna*^F/F^ mouse cardiac myocytes were associated with changes in the localization of ∼58% of the DEGs, and the CTCF motifs had the largest effect on the expression of the DEGs, whereas changes in the chromatin compartments and TADs did not affect the transcript levels of the DEGs in the LMNA-deficient cardiac myocytes. The findings also suggest that CTCF affects gene expression independent of its function in loop formation.

Stringent conditions were used during data analysis. For example, recurring DSBs, i.e. present in at least two samples and not the singletons, were used to determine the association of CTCF, chromatin compartments, TADs, and loops with DSBs. Likewise, for the Hi-C data, TADs and loops that were present in two out of three independent samples were used in the data analysis. The CTCF peaks were analyzed both as invariants and as a whole, and only cLADs that were present in three out of five samples were analyzed. Similarly, gain and loss of loops were consistently observed among the three biological replicates in each genotype. Overall, the data were consistent across multiple analyses, showing the contributions of CTCF, TADs, and loops to the DEGs in *Myh6-Cre:Lmna*^F/F^ mouse cardiac myocytes. The findings, by and large, are associative in nature and would require further validation through replication and experimentation.

In conclusion, LMNA deficiency is associated with modest changes in chromatin compartments but considerable changes in the genome-wide CTCF binding sites and organization of TADs and loops in cardiac myocytes. The chromatin remodeling is associated with dysregulated gene expression and increased prevalence of DSBs in cardiac myocytes. These findings are consistent with, but do not prove, a role for altered CTCF occupancy and loop architecture in transcriptional dysregulation in cardiac myocytes. Collectively, the findings provide insights into the phenotypic diversity and molecular pathogenesis of cardiac involvement in laminopathies.

## Supplementary Material

ugag046_Supplemental_File

## Data Availability

All data are available from the corresponding authors upon request. The RNA-Seq, CUT&RUN, and Hi-C data have been deposited at GEO (RNA-Seq: GSE309726, END-Seq: GSE250321, LMNA CUT&RUN: GSE250321, CTCF CUT&RUN and Hi-C: GSE329699.
