# Supplementary material for: Lamin A regulates chromatin structure and function in cardiac myocytes in a mouse model of lamin-cardiomyopathy
Source: NAR Mol Med. 2026 Oct 22;3(4):ugag046. doi: 10.1093/narmme/ugag046 (PMC13748967; doi:10.1093/narmme/ugag046)
Supplement: Supplementary file 1 [file ugag046_supplemental_file.pdf]

Wild type cardiac myocyte genomes

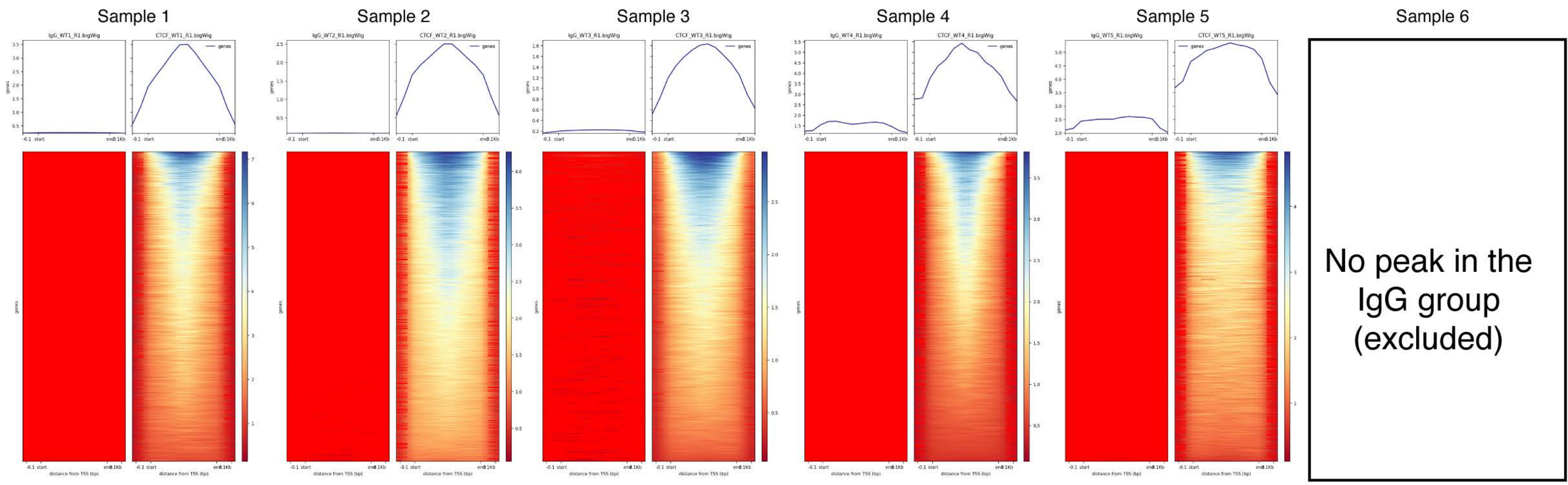

Myh6-Cre-Lmna$^{F/F}$ cardiac myocyte genomes

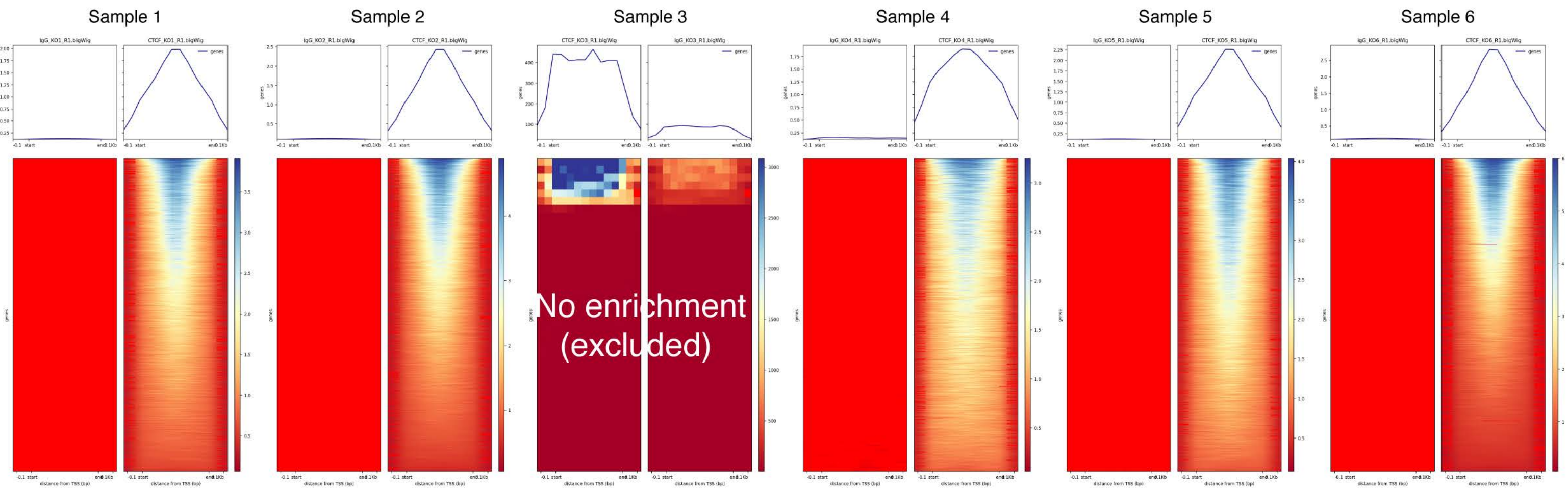

Online Figure 1. Heat maps of the peaks detected in each sample with the anti-CTCF antibody as compared to the corresponding IgG in the wild type (upper panel) and *Myh6-Cre:Lmna*$^{F/F}$ (lower panel) cardiac myocyte genomes. Sample # 6 in the wild type group had a very low number of CTCF peaks in the anti-CTCF antibody group and sample # 3 in the *Myh6-Cre:Lmna*$^{F/F}$ group had a very low peak in the IgG group. To avoid the confouding effects of these technical problems, these two samples were excluded from the subsequent analysis.

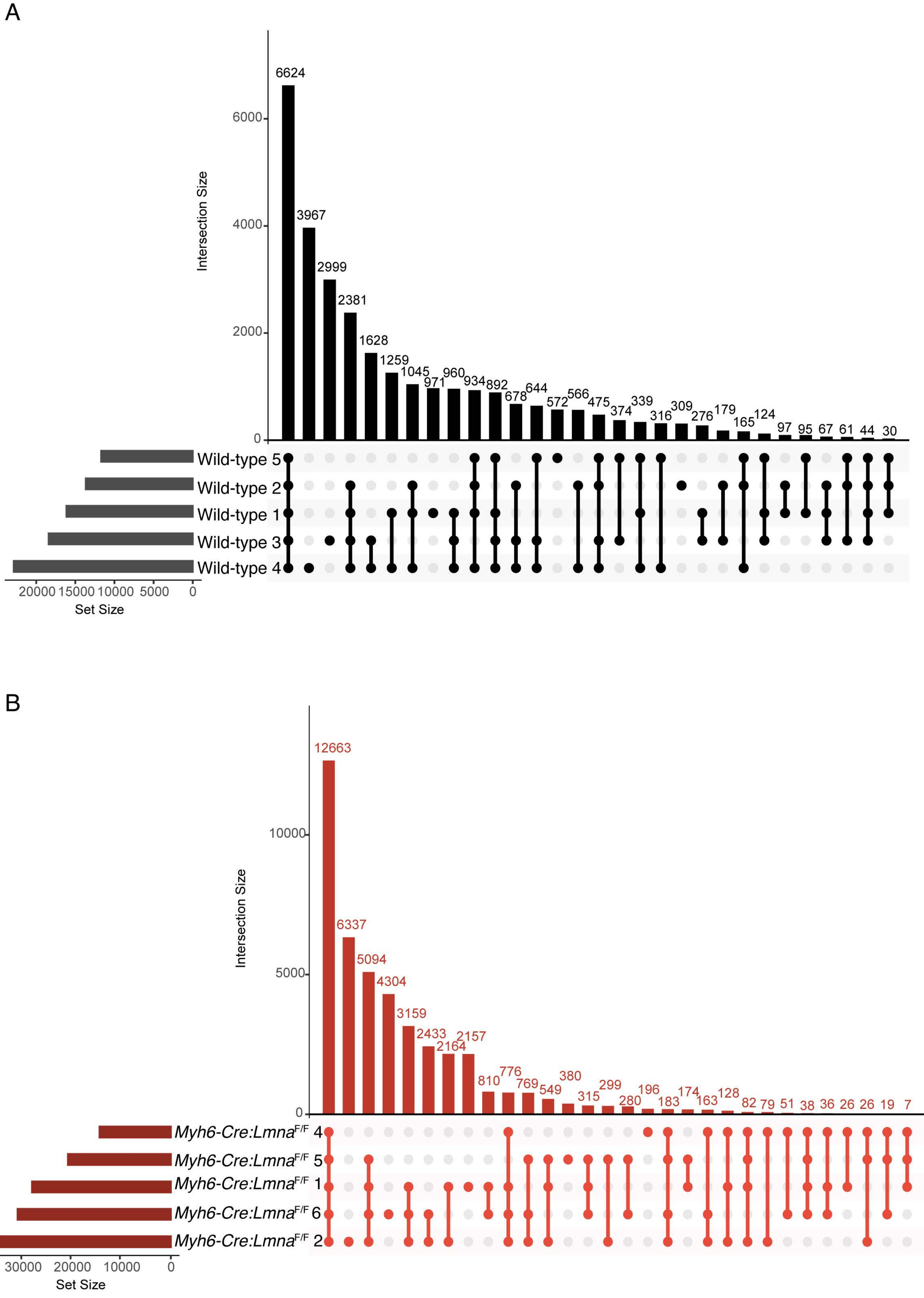

Online Figure 2. Overlap of the CTCF peaks in the wild type (A) and *Myh6-Cre-Lmna*$^{F/F}$ (B) cardiac myocyte genomes, depicting singleton, shared, and invariants peaks among the samples.

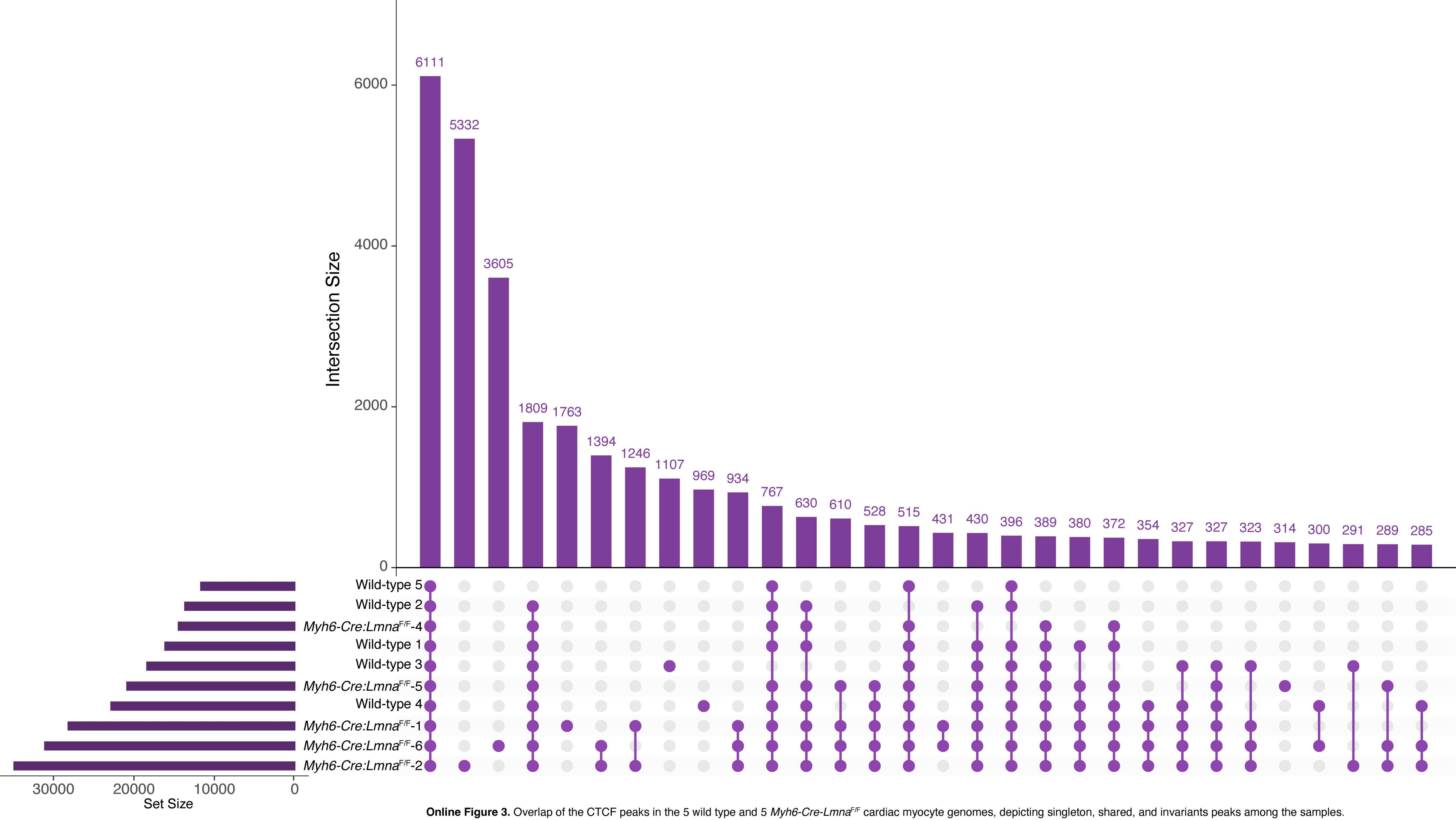

Online Figure 3. Overlap of the CTCF peaks in the 5 wild type and 5 *Myh6-Cre-Lmna*$^{F/F}$ cardiac myocyte genomes, depicting singleton, shared, and invariants peaks among the samples.

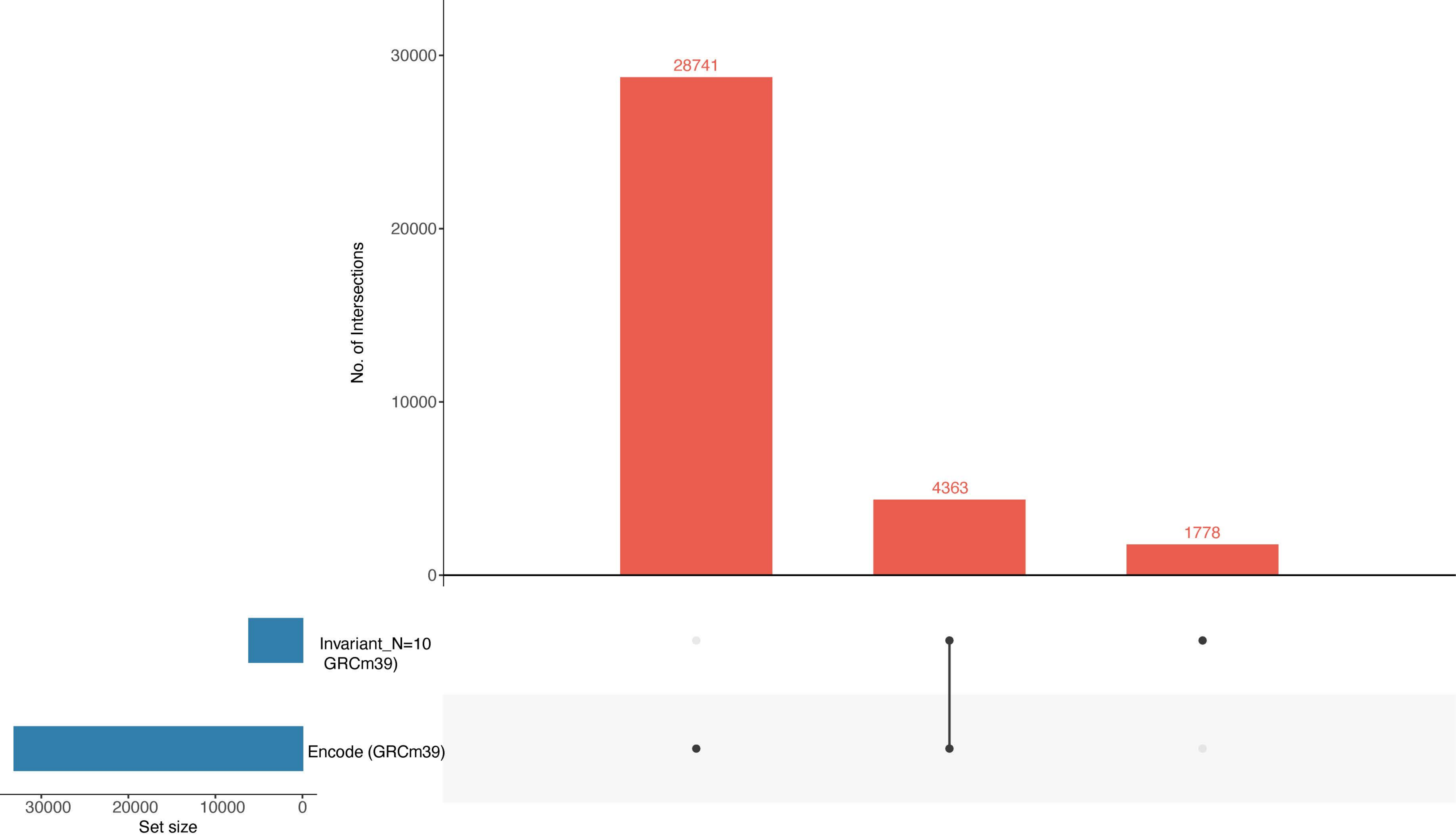

Online Figure 4. Intersection of the CTCF peaks in cardiac myocyte genomes with the CTCF peaks in the Encode dataset.

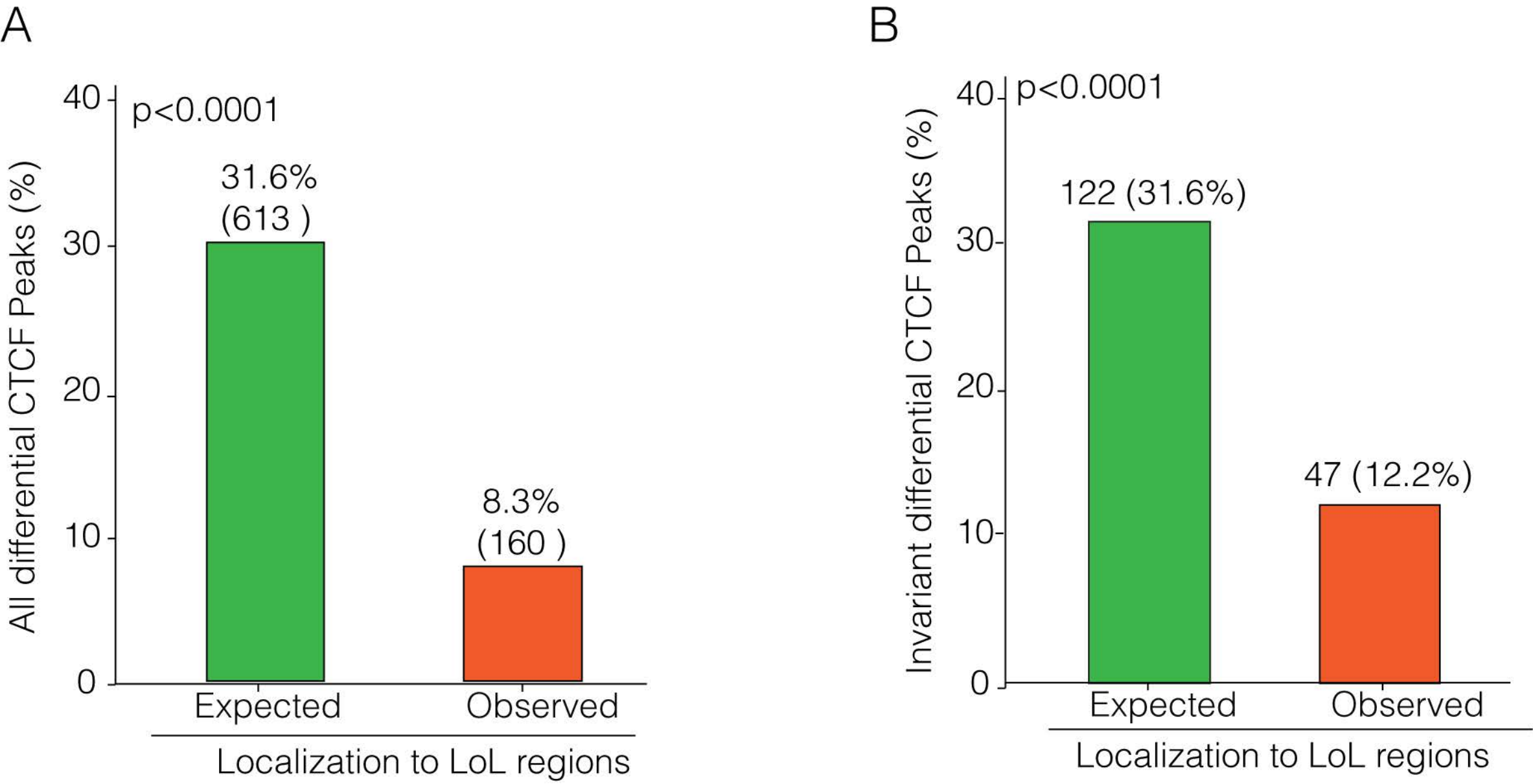

Online Figure 5. Localization of the differential CTCF peaks to the loss of LAD (LoL) regions.

A. All differential CTCF Peaks between the WT and *Myh6-Lmna*$^{F/F}$ cardiac myocytes
B. The differential CTCF Peaks that were invariant between the two genotypes

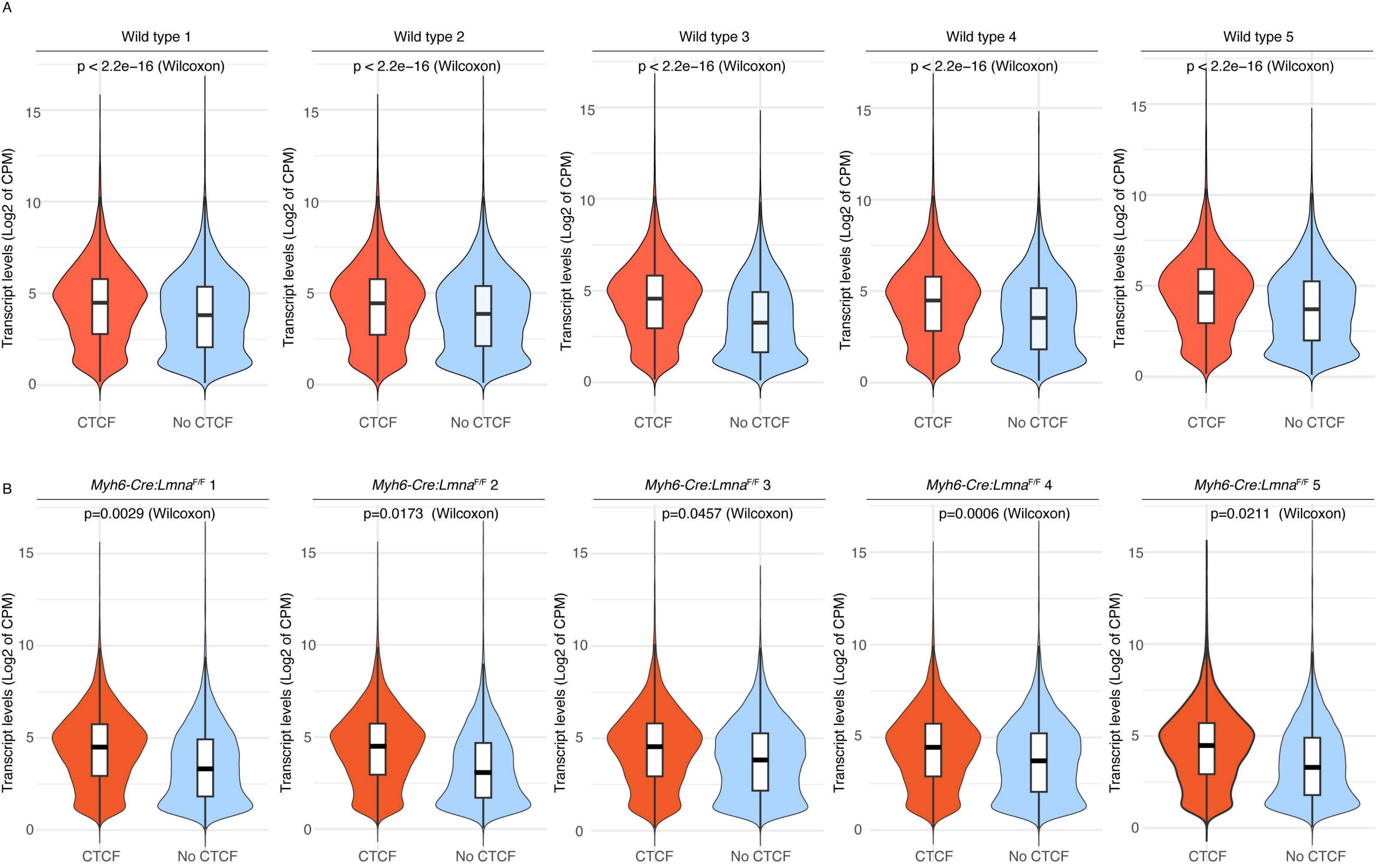

Online Figure 6. Association of the CTCF motifs (±2 kb of TSS) with gene expression. Transcript levels of genes whose TSSs contaiend at least one CTCF motif within 10 Kbp proximally were compoared to the transcript levels of genes that did not contain a CTCF motif within 10 kbp of their TSS. The differences were analyzed by the non-parametic Wilcoxon rank test between the two groups in each mouse.

A. Wild type mice. **B.** *Myh6-Cre:Lmna*$^{F/F}$ mice.

Because in each mouse CTCF motifs are located at different genomic sites the group effects were analyzed only for the invariant CTCF sites and the results are presented in the main figure.

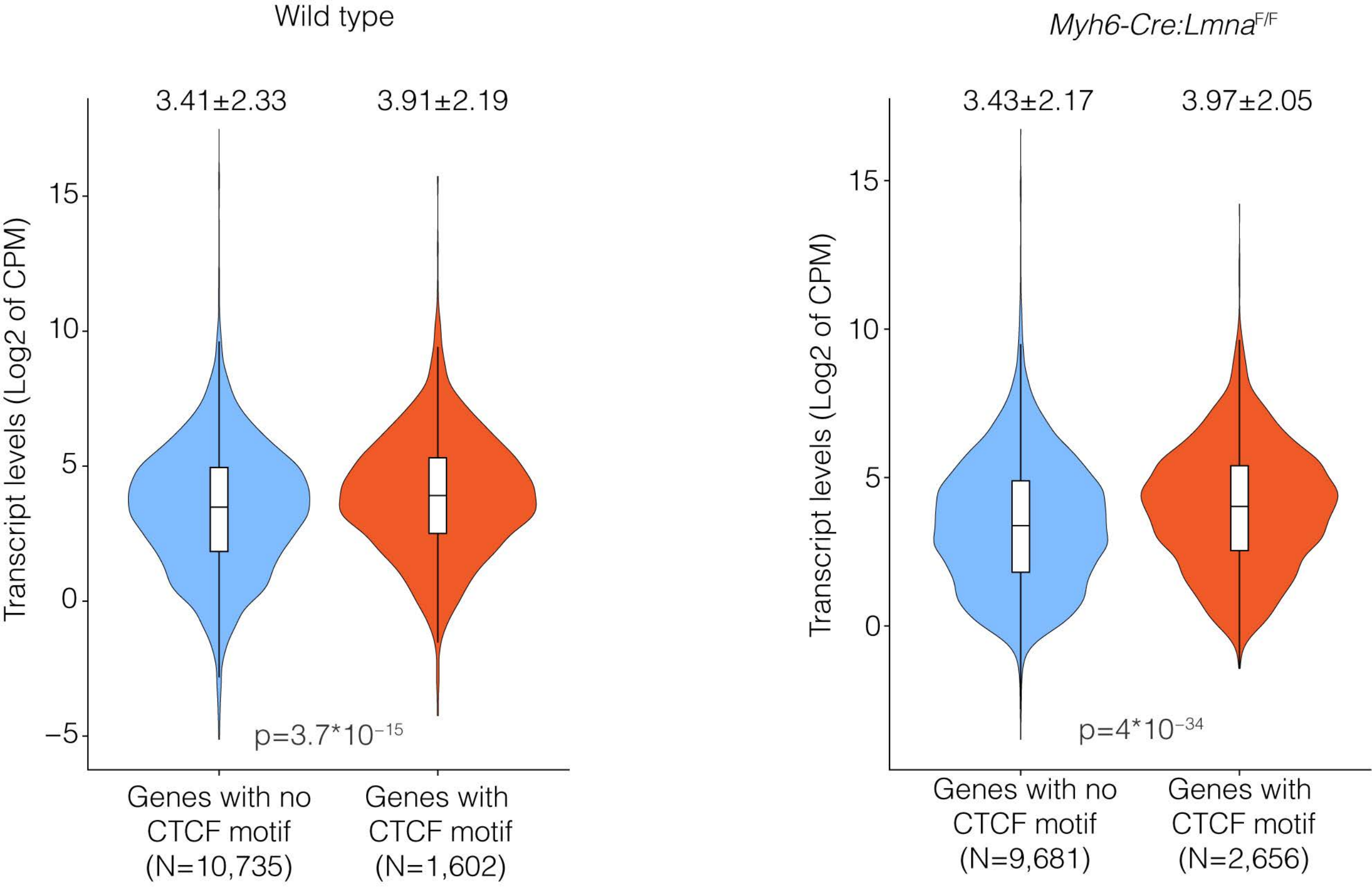

Online Figure 7. Transcript levels of genes containing or lacking CTCF motif in the wild-type and *Myh6-Cre:Lmna*$^{F/F}$ cardiac myocytes.

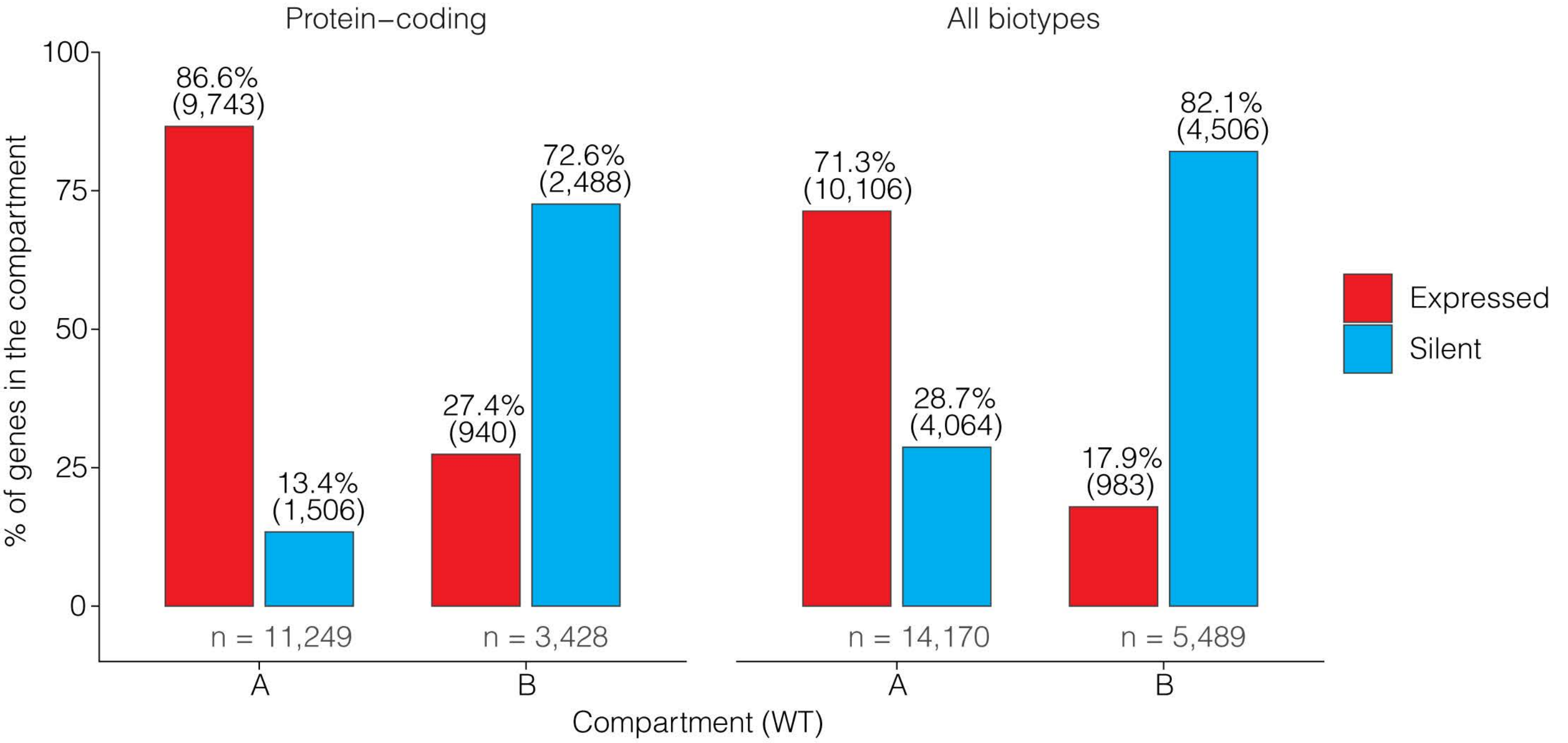

Online Figure 8. Frequency of expressed and silent protein coding and all biotypes located in compartments A and B (Genes with transcript levels below one CPM in any of the samples were excluded).

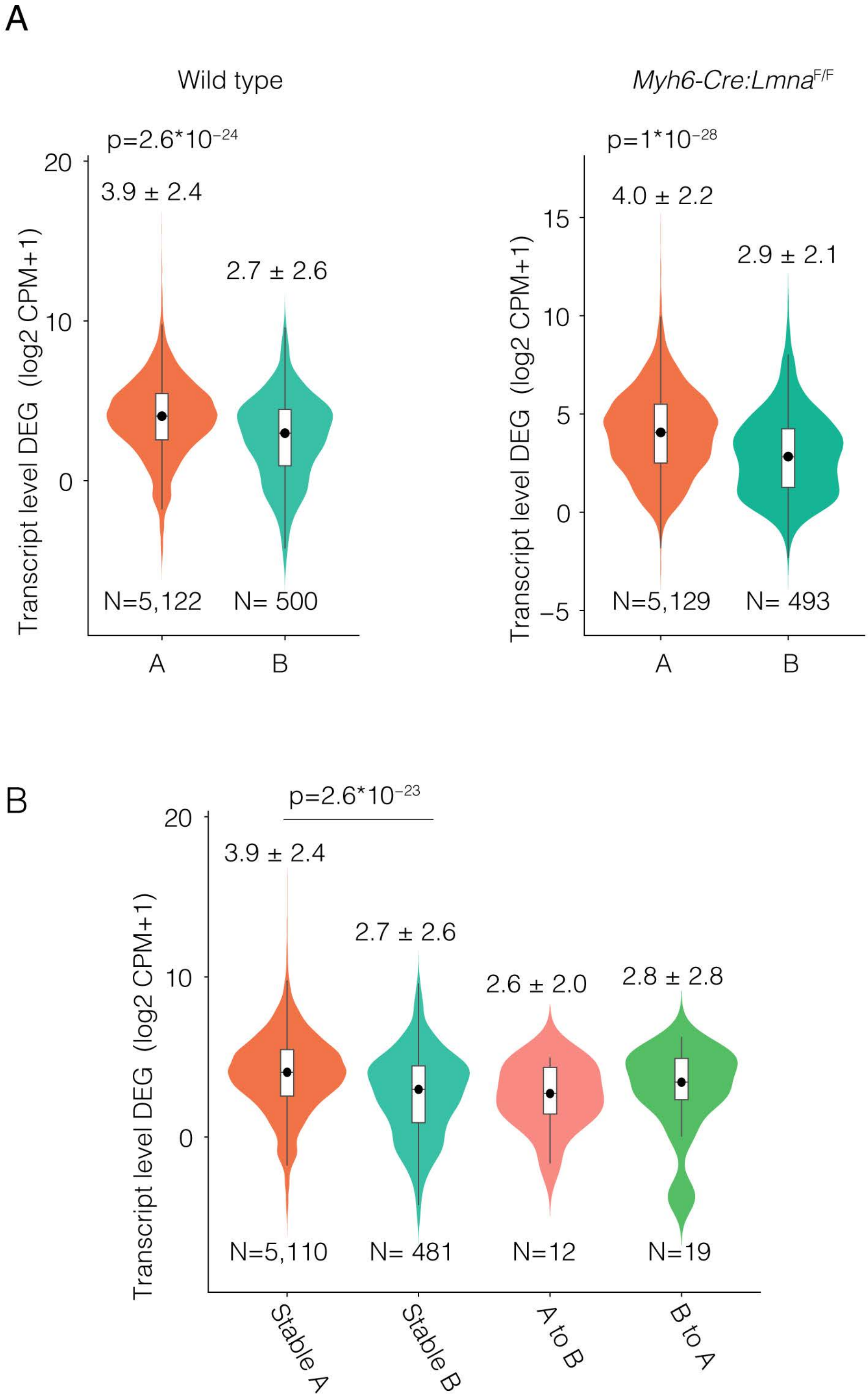

Online Figure 9. Effects of compartmental switch on gene expression in the LMNA-deficient cardiac myoctytes

A. Transcript levels of the differentially expressed genes in compartment A and B in the wild-type and *Myh6-Cre:Lmna*$^{F/F}$ cardiac myocytes.

B. Transcript levels of the differentially expressed genes located in stable A and B compartments and those in compartments that were switched from A to B and B to A.

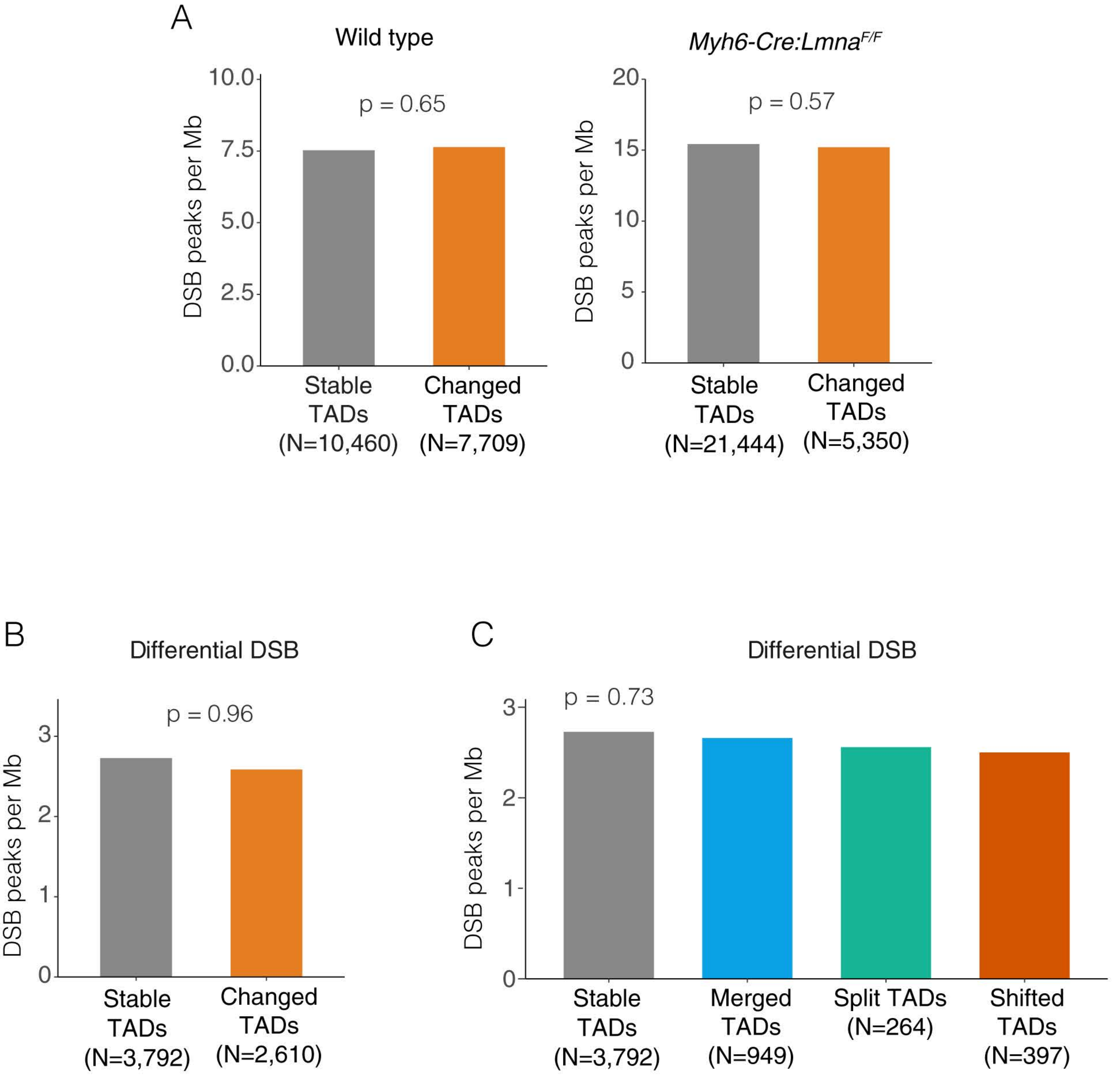

Online Figure 10. Shift in TADs and DSBs in cardiac myocytes.

A. Prevalence of DSBs at the sites of stable and changed TADs in the wild-type and *Myh6-Cre:Lmna*$^{F/F}$ cardiac myocyte genomes.

B. Prevalence of differential DSBs at the sites of stable and changed TADs

C. Prevalnces of of the differential DSBs at the sites of stable and merged, split and shifted TADs.

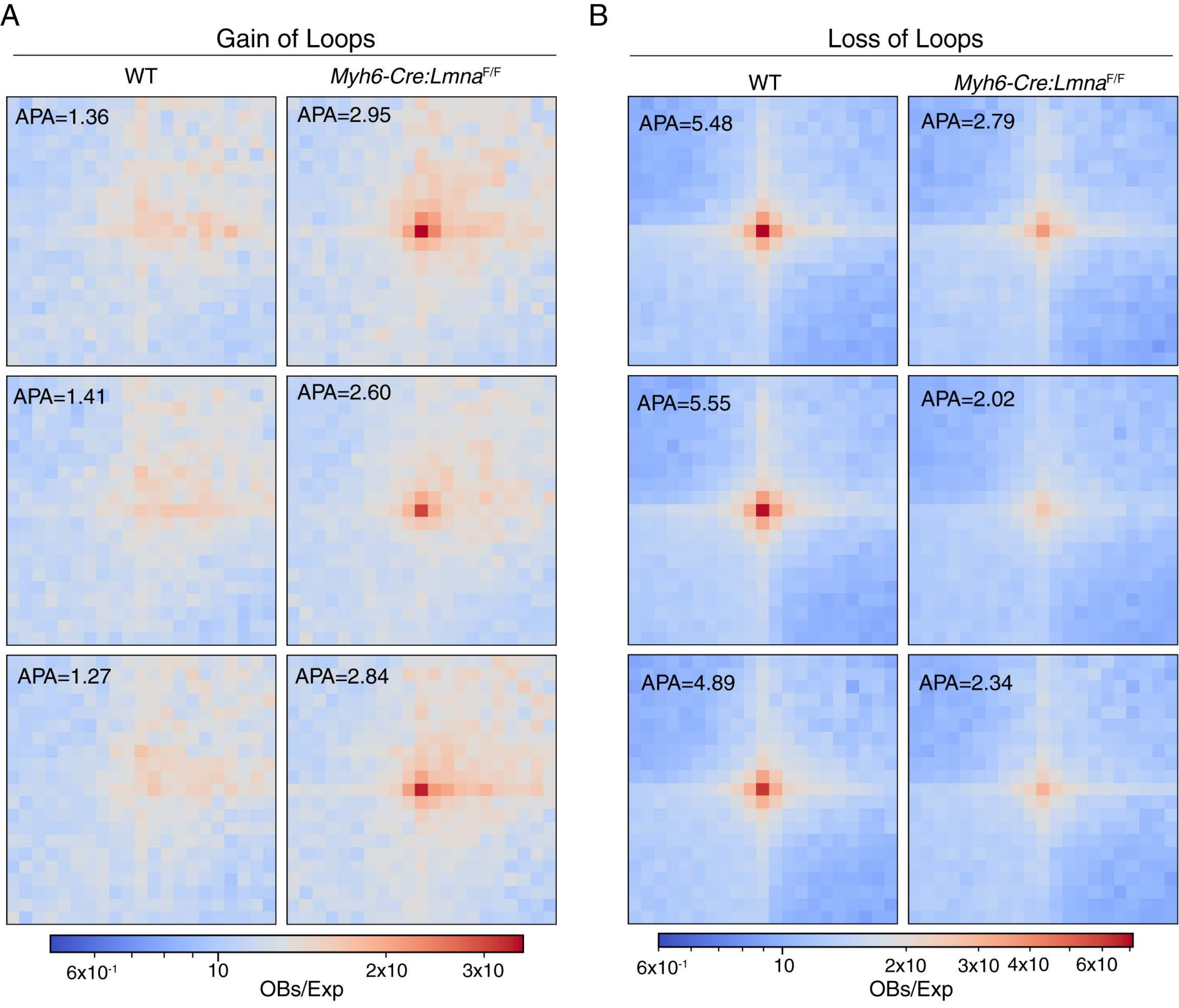

Online Figure 11. Consistency of chromatin loop calling among the samples in the WT and *Myh6-Cre:Lmna*$^{F/F}$ genotypes.

A. Aggregate loop analysis (APA) in each sample per genotype for the gain of loops.
B. Aggregate loop analysis for each sample per genotype for the loss of loops.

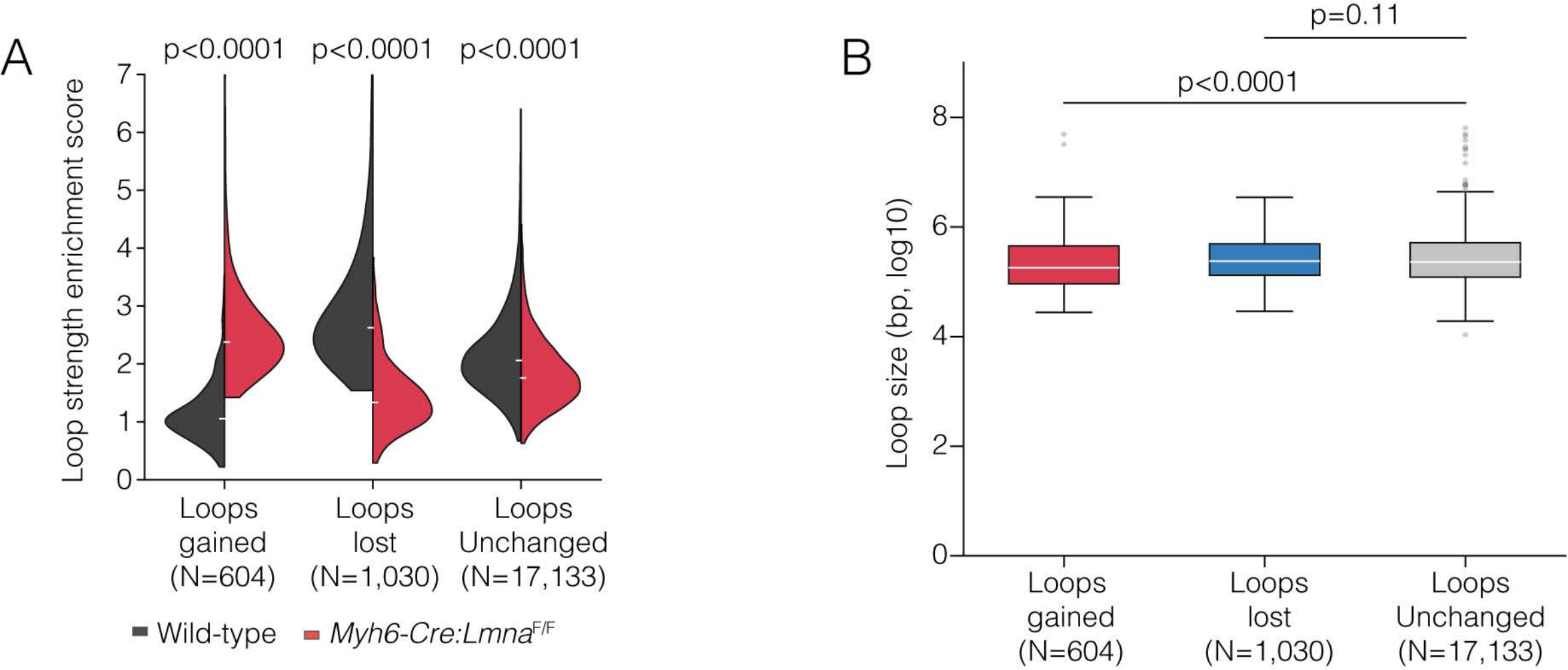

Online Figure 12. Loops characteristics

A. Loop strength enrichment in the wild-type and *Myh6-Cre:Lmna*F/F myocyte genomes.

B. Loop size in the loops gain. lopps lost, and loops unchanged. Median and range are shown.

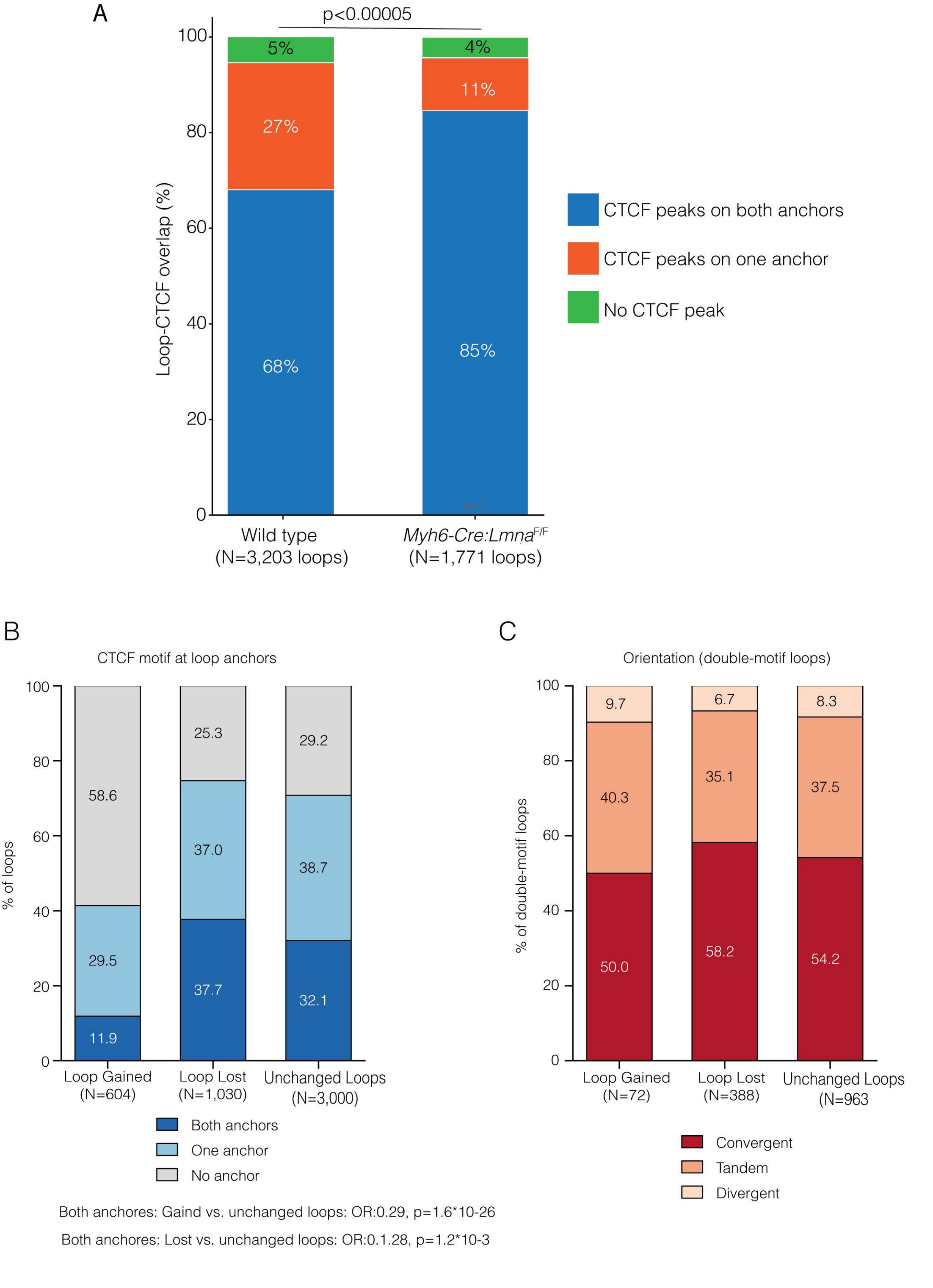

Online Figure 13. CTC F Peaks and loop restructuring

A. Localization of CTCF Peaks at loop anchores in the wild-type and *Myh6-Cre:Lmna*$^{F/F}$ myocyte genomes

B. Localization of CTCF peaks at the loop gained, lost,. or unchanged regions shown as percentage with CTCF peaks at both, one or no anchores.

C. Percentage double CTCF peak patterns at the loop gained, lost, or unchanged regions.

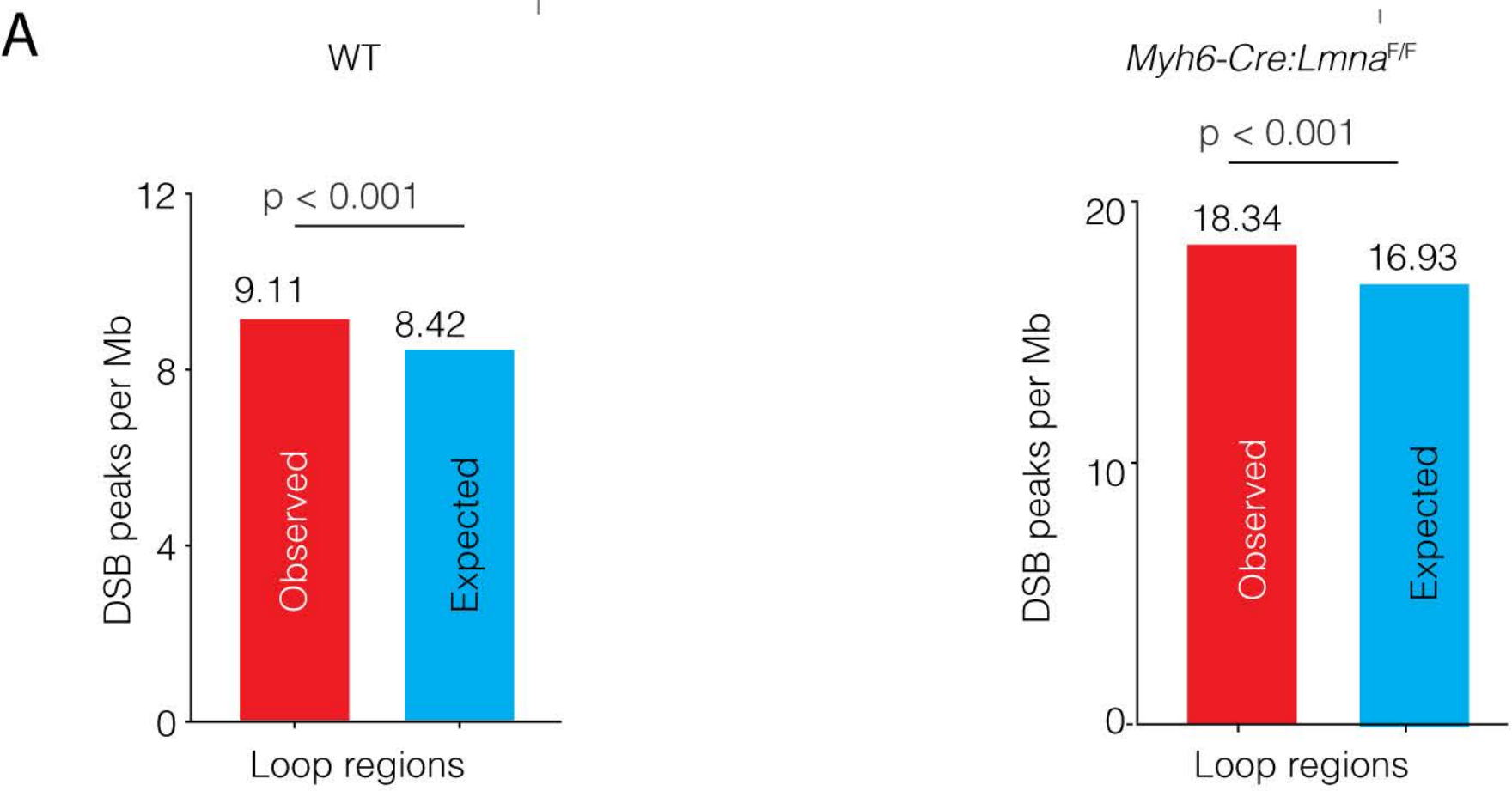

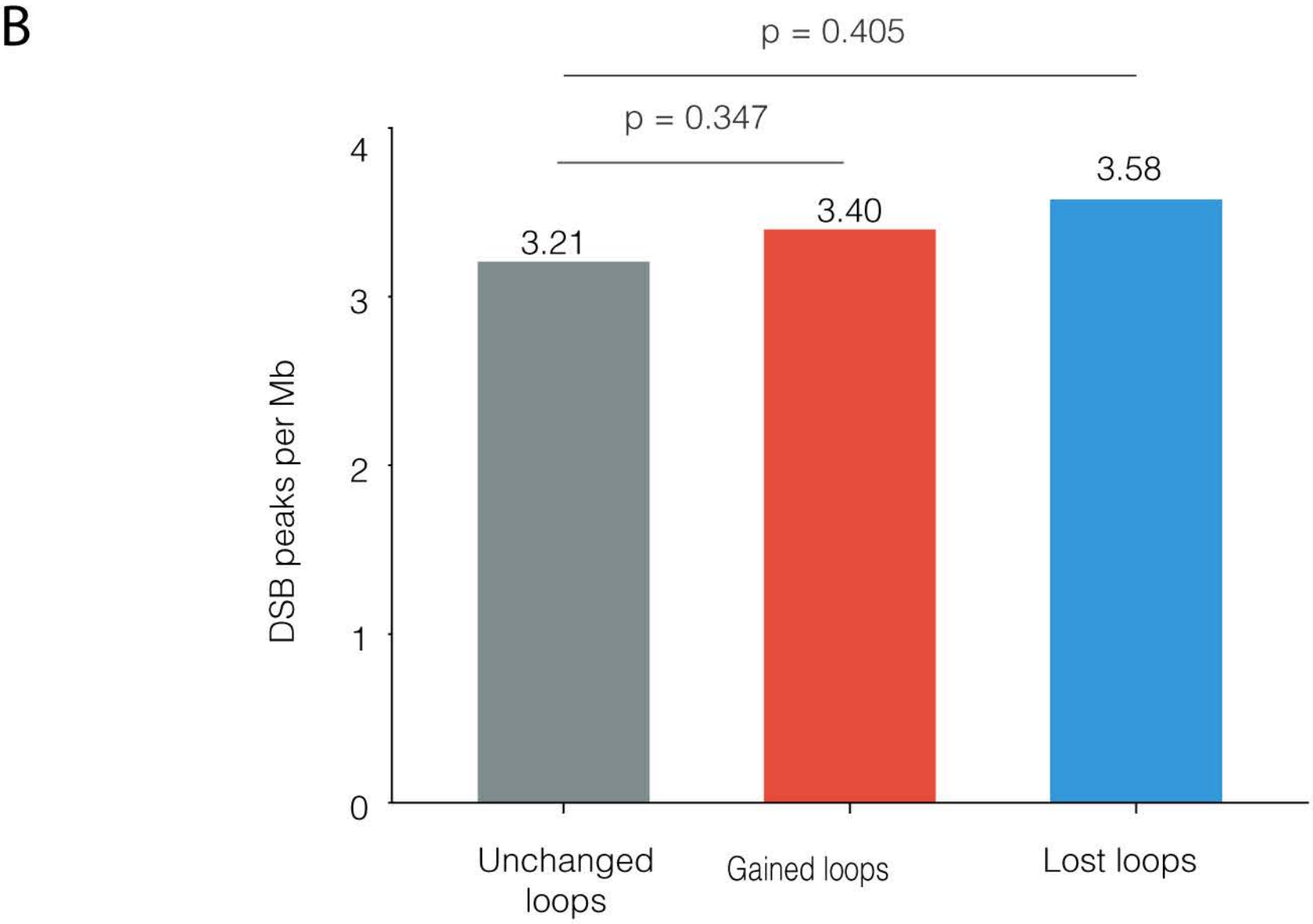

Online Figugre 14. Prevalence of DSBs at chromatin loop regions.

A. Observed and expected frequencies of DSBs at the loops regions in the wild-type and *Myh6-Cre:Lmna*$^{F/F}$ myocyte genomes.

B. DSB denstiy at the unchanged loop, gained loop, and lost loop regions.

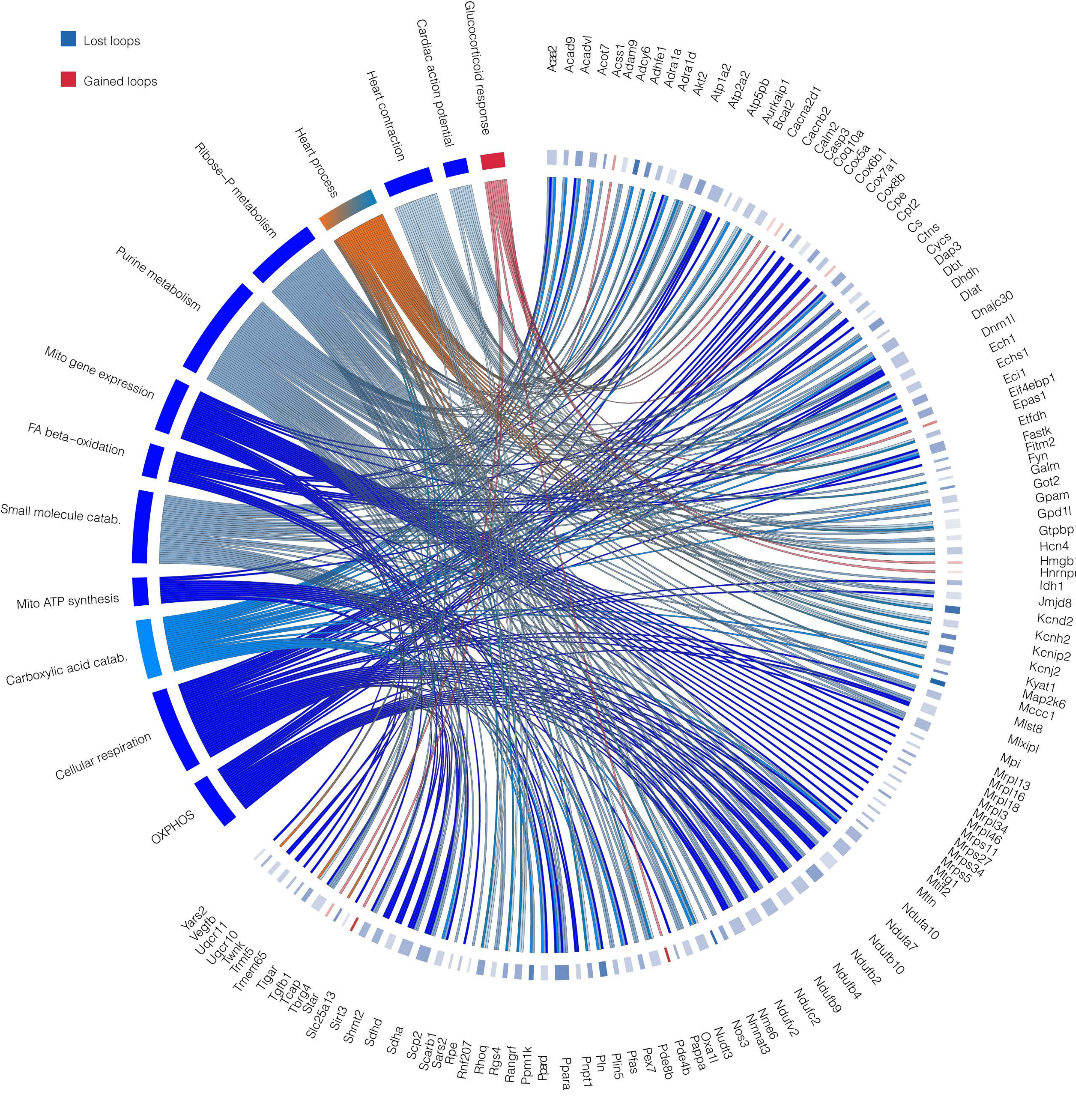

Online Figure 15. Effects of chromatin loop reorganization on biological pathways

Online Table 1

Terminology and key abbreviations

LADs: Lamin-associated domains

TADs: Topologically-associated domains

CTCF: CCCTC-binding factor

Invariant CTCF peaks: Peaks that were present in all samples, regardless of the genotype

Invariant CTCF peaks in the wild-type genome: CTCF Peaks that were present in all 5 independent samples from wild -type mice

Invariant CTCF peaks in *Myh6-Cre:Lmna*$^{F/F}$ genome: CTCF Peaks that were present in all 5 independent samples from *Myh6-Cre:Lmna*$^{F/F}$ mice.

Differential CTCF variants: CTCF peaks that reached statistical significance when comparing signaling intensity between the WT and *Myh6-Cre:Lmna*$^{F/F}$ genomes.

Consensus TAD or loop peaks: Peaks that were present in at least 2 out of the 3 independent samples in the HiC dataset.

Online Table 2. Quality control metrics in the CTCF CUT&RUN assays

	**Total paired reads (M)**		**Mapped paired reads (%)**		**Mapped paired reads (M)**		**Peaks (N)**	**Average Peak length (bp)**	
**Genotype**	**IgG**	**CTCF antibody**	**IgG**	**CTCF antibody**	**IgG**	**CTCF antibody**		**Average (bp)**	**SD**
**WT-1**	7.26783311	25.7208766	74.3	86.7	5.4	22.3	16511	313.22	142.87
**WT-2**	22.2936763	15.7397692	93.3	95.3	20.8	15	13889	374.27	160.39
**WT-3**	27.6617954	56.2105263	95.8	95	26.5	53.4	18951	324.36	169.42
**WT-4**	12.7173913	33.2615716	92	92.9	11.7	30.9	23441	342.37	181.25
**WT-5**	10.3864734	19.4413408	82.8	89.5	8.6	17.4	12020	290.81	139.93
**WT-6**	**0.11235955**	**31.4911366**	**89**	**95.9**	**0.1**	**30.2**	**0**	**0.00**	**0.00**
**Mean (excluding sample 6)**	**16.0654339**	**30.0748169**	**87.64**	**91.88**	**14.6**	**27.8**	**16962.4**	**329.0047514**	**158.775096**
**SD (excluding sample 6)**	**8.57502718**	**16.0526627**	**8.93045352**	**3.70702037**	**8.79062**	**15.5500804**	**4472.23812**	**31.45356972**	**17.5279945**
***Myh6-Cre:Lmna*$^{F/F}$-1**	13.258427	35.4143019	89	88.1	11.8	31.2	28893	359.96	201.89
***Myh6-Cre:Lmna*$^{F/F}$-2**	19.1061453	34.6491228	89.5	91.2	17.1	31.6	35456	395.48	231.26
***Myh6-Cre:Lmna*$^{F/F}$-3**	18.2232346	28.0182232	87.8	87.8	**16**	**24.6**	34	406.00	870.17
***Myh6-Cre:Lmna*$^{F/F}$-4**	19.4776931	27.4211099	91.9	91.9	17.9	25.2	14822	329.21	159.32
***Myh6-Cre:Lmna*$^{F/F}$-5**	15.7629256	35.8381503	79.3	86.5	12.5	31	21226	384.71	187.77
***Myh6-Cre:Lmna*$^{F/F}$-6**	18.5810811	28.6804798	88.8	91.7	16.5	26.3	31409	460.08	290.31
**Mean (excluding Sample 3)**	**17.2372544**	**32.400633**	**87.7**	**89.88**	**15.16**	**29.06**	**26361.2**	**385.8883922**	**214.110106**
**SD (excluding sample 3)**	**2.66096327**	**4.01838344**	**4.85643902**	**2.43556975**	**2.80321244**	**3.05417747**	**8278.42223**	**48.68790977**	**49.8641007**

Abbreviations: WT: wild type; M: million; N: Number; bp: base pair; CTCF: CCCTC-binding factor; IgG: Immunoglobulin G; *Myh6-Cre:Lmna*$^{F/F}$: deletion of the floxed allele of the *Lmna* gene in cardiac myocytes; SD: standard deviation.

Red color indicates samples that were removed from the data analysis due to technical problems.

Online Table 3

Genomic Localization of the CTCF Peaks

A. Genomic Localization of Invariant CTCF Peaks (Present in all 10 samples)

Feature	Number	Obs %	Exp %	Ratio	p-value	p-Bonf
Promoter-TSS	1107	18.115	1.185	15.283	0.00E+00	0.00E+00
5' UTR	77	1.260	0.089	14.086	1.31E-59	1.05E-58
Exon	155	2.536	1.314	1.93	9.64E-14	7.71E-13
3' UTR	95	1.555	0.785	1.981	1.68E-09	1.35E-08
Intron	1948	31.877	35.955	0.887	2.16E-11	1.72E-10
TTS	200	3.273	1.072	3.054	2.61E-41	2.09E-40
Non-coding	43	0.704	0.166	4.251	1.30E-14	1.04E-13
Intergenic	2486	40.681	59.411	0.685	9.77E-191	7.82E-190

B. Genomic Localization of Singleton CTCF Peaks (Present only in 1 Samples)

Feature	Number	Obs %	Exp %	Ratio	p-value	p-Bonf
Promoter-TSS	1829	13.294	1.185	11.216	0.00E+00	0.00E+00
5' UTR	167	1.214	0.089	13.57	1.54E-124	1.23E-123
Exon	465	3.380	1.314	2.572	7.19E-71	5.75E-70
3' UTR	153	1.112	0.785	1.417	3.83E-05	3.06E-04
Intron	6262	45.515	35.955	1.266	4.13E-117	3.30E-116
TTS	276	2.006	1.072	1.872	1.64E-21	1.31E-20
Non-coding	87	0.632	0.166	3.82	8.84E-25	7.07E-24
Intergenic	4519	32.846	59.411	0.553	0.00E+00	0.00E+00

Online Table 4

Genomic Localization of the Differential CTCF Peaks

A. Genomic localization of invariant CTCF Peaks that were differential between the two genotypes

Feature	Number	Obs %	Exp %	Ratio	p-value	p-Bonf
Promoter-TSS	56	14.545	1.201	12.107	7.48E-42	5.98E-41
5' UTR	2	0.519	0.090	5.743	4.82E-02	3.85E-01
Exon	7	1.818	1.334	1.363	3.69E-01	1.00E+00
3' UTR	2	0.519	0.798	0.651	7.75E-01	1.00E+00
Intron	148	38.442	36.513	1.053	4.28E-01	1.00E+00
TTS	10	2.597	1.086	2.392	1.04E-02	8.30E-02
Non-coding	3	0.779	0.168	4.647	2.77E-02	2.22E-01
Intergenic	157	40.779	58.786	0.694	1.59E-12	1.27E-11

B. Genomic localization of all differential CTCF peaks between the two genotypes

Feature	Number	Obs %	Exp %	Ratio	p-value	p-Bonf
Promoter-TSS	94	4.848	1.201	4.035	8.38E-29	6.71E-28
5' UTR	6	0.309	0.090	3.421	9.19E-03	7.35E-02
Exon	24	1.238	1.334	0.928	8.43E-01	1.00E+00
3' UTR	21	1.083	0.798	1.358	1.59E-01	1.00E+00
Intron	963	49.665	36.513	1.36	4.12E-32	3.29E-31
TTS	52	2.682	1.086	2.47	8.08E-09	6.46E-08
Non-coding	9	0.464	0.168	2.768	6.26E-03	5.01E-02
Intergenic	770	39.711	58.786	0.676	7.55E-64	6.04E-63

Online Table 5

Localization of the CTCF Peaks to cLAD regions

**A. Localization of the Invariant CTCF Peaks (shared in 5 samples) to the cLAD Regions in the Wild Type Mouse Cardiac Myocyte Genome** (mm39)	
Total CTCF motifs (MA0139.1)(N)	146,366
Total CTCF peaks (N)	6,624
Peaks inside cLADs (N)	786
CTCF peaks inside cLADs (%)	11.87
Peaks outside cLADs (N)	5,838
CTCF peaks outside cLADs (%)	88.13
Total cLADs (bp)	831,494,249
Mouse genome size (bp, std chr)	NA
Total cLADs (% of the genome)	31.32
Expected CTCF peaks in cLADs (N, if random)	2,075
Expected CTCF peaks in cLADs (%, if random)	31.32
Observed / Expected CTCF peaks in cLADs (fold enrichment)	0.379
Fold depletion (1 / 0.379)	2.64-fold
Odds ratio	0.29
Fisher exact p value	<0.0001

**B. Localization of All CTCF Peaks (pooled in 5 samples) to the cLAD Regions in the Wild Type Mouse Cardiac Myocyte Genome** (mm39)	
Total CTCF peaks (MA0139.1)(N)	146,366
Total CTCF peaks (N)	29,786
Peaks inside cLADs (N)	3,874
CTCF peaks inside cLADs (%)	13.01
Peaks outside cLADs (N)	25,912
CTCF peaks outside cLADs (%)	86.99
Total cLADs (bp)	831,494,249
Mouse genome size (bp, std chr)	NA
Total cLADs (% of the genome)	31.32
Expected CTCF peaks in cLADs (N, if random)	9,330
Expected CTCF peaks in cLADs (%, if random)	31.32
Observed / Expected CTCF peaks in cLADs (fold enrichment)	0.415
Fold depletion (1 / 0.415)	2.41-fold
Odds ratio	0.33
